# Novel Venetin-1 nanoparticle from earthworm coelomic fluid as a promising agent for the treatment of non-small cell lung cancer

**DOI:** 10.1038/s41598-022-21665-8

**Published:** 2022-11-02

**Authors:** Magda Rybicka, Paulina Czaplewska, Jolanta Rzymowska, Weronika Sofińska-Chmiel, Sylwia Wójcik-Mieszawska, Kinga Lewtak, Katarzyna Węgrzyn, Przemysław Jurczak, Agata Szpiech, Jakub Nowak, Natalia Musiał, Marta J. Fiołka

**Affiliations:** 1 Intercollegiate Faculty of Biotechnology of University of Gdańsk and Medical University of Gdańsk, Gdańsk, Poland; 2 Department of Biology and Genetics, Medical University of Lublin, Lublin, Poland; 3 Analytical Laboratory, Institute of Chemical Sciences, Faculty of Chemistry, Maria Curie-Skłodowska University, Lublin, Poland; 4 Department of Immunobiology, Institute of Biological Sciences, Maria Curie-Skłodowska University, Lublin, Poland; 5 Department of Cell Biology, Institute of Biological Sciences, Maria Curie-Skłodowska University, Lublin, Poland; 6 Faculty of Chemistry, University of Gdańsk, Gdańsk, Poland; 7 Malopolska Centre of Biotechnology, Jagiellonian University, Kraków, Poland

**Keywords:** Biotechnology, Cancer, Cell biology, Drug discovery, Oncology

## Abstract

The present research shows the antitumor activity of a protein-polysaccharide complex Venetin-1 obtained from the coelomic fluid of *Dendrobaena veneta* earthworms against A549 cancer cells. The investigations are a continuation of experiments on the antitumor activity of coelomic fluid obtained from this species. The Venetin-1 nanoparticle was obtained after thermal treatment of the coelomic fluid, separation from coelomocytes, filtration, and lyophilization. The preparation showed a selective effect on cancer cells, whereas normal cells were unaffected. Venetin-1 was effective against the lung cancer cells at doses of 31.3 and 62.5 µg/ml, and the results were imaged using light microscopy and scanning electron microscopy (SEM). The cells died mainly via the apoptosis pathway. Necrotic cells appeared sporadically in the microscopic view. SEM imaging revealed complete destruction of the A549 cells after the incubation with Venetin-1. The atomic force microscopy (AFM) analyses showed changes in the topography, peak force error images, and Young’s modulus (elasticity) of the A549 cells after the incubation with Venetin-1. The transmission electron cryomicroscopy (Cryo-TEM) analysis indicated a polymeric nature of the analyzed preparation. The samples of Venetin-1 showed a very homogeneous size profile with the microparticle size of approximately 58.23 nm. A significant decrease in Venetin-1 binding to sphingomyelin was observed. Venetin-1 lost its pore-forming activity or deactivation of the pore-forming activity occurred. This confirms the absence of hemolytic capacity of Venetin-1 towards red blood cells. The conducted analyses show the suitability of the obtained complex for biomedical research. The next step will consist in analyses of the effect of Venetin-1 on the immune system in mice.

## Introduction

In the group of non-communicable disease, cancers are the most common causes of death. Lung cancer is characterized by the highest mortality among men and woman worldwide^1,2^. Cigarette smoking is the most common cause of lung cancer development. Tobacco smoke contains about 4000 substances, with approximately 50 compounds classifieds as toxic, irritating, or carcinogenic^1,3,4^. Individuals exposed to passive smoking are at risk of lung cancer as well. The metabolites of nicotine can be detected in passive smoker’s urine, which indicates that non-smokers inhale various components of tobacco smoke^3^. Tobacco smoke components cause disorders in the cell genome, e.g., DNA deletion or amplification, incorrect methylation, and even loss or gain of whole chromosomes^2^.

Although 85% of lung cancers develop in tobacco smokers, the other cases are diagnosed in those who have never smoked. One of the non-tobacco causes of lung cancers is air pollution, predominantly the presence of sulfur oxygen, nitrogen oxygen, or dust with a diameter less than 2.5 µm^5,6^. In the US, the leading cause of lung cancer is radon, i.e., the product of radium decay present in soil. This gas is the cause of 40% of cancer deaths, and most of these patients are lifetime non-smokers^1^. Researchers have also identified genes responsible for the susceptibility to lung cancer development, i.e., germline mutations in the *p53* and *EFGR* genes, SNP gene polymorphism, or disorders in DNA repair genes, e.g. *ERCC1*^2,7^.

Lung cancers are diagnosed in people aged about 70 years. They can be classified into two main types: SCLC (Small Cell Lung Carcinoma) and NSCLC (Non-Small Cell Lung Carcinoma). Approximately 85% of lung cancers are NSCLC^2^. They often have poor prognosis due to the late detection and advanced stage of cancer development^8^. SCLCs are often located in larger airways, causing bronchial occlusions. These cancers are often quite big and often metastasize to lymph nodes^9^.

It has been documented that many compounds of natural plant, fungal, or animal origin can destroy lung cancer cells. For example, Asian basil extract from *Ocimum sanctum* exhibited cytotoxicity to A549 lung cancer cells, increased the sub-G1 population, and influenced the appearance of apoptotic bodies in cancer cells^10^. Other studies showed that *Phyllanthus* was able to induce selective toxicity on A549 cells. Moreover, it exerted an inhibitory effect at the critical stages of metastasis, including migration and invasion^11^. In turn, Ramalakshmi et al.^12^ found cytotoxic activity of ethanol extract from *Coleus amboinicus* leaves against A549 lung cancer cells. Its cytotoxicity after 72 h of incubation with A549 lung cancer cells was 94%.

Wei-Sheng et al.^13^ showed that bostricin (hydroxy-methoxy-tetrahydro-5-methylanthracene) from marine fungi inhibited proliferation of A549 cells in a dose- and incubation time-dependent manner, arrested the cell cycle at G0/G1, and stimulated apoptosis. Pycnidione isolated from the *Theissenia rogersii* fungus was shown to reduce the proliferation of A549 lung adenocarcinoma cells via cell cycle arrest at the G1 phase. Moreover, it was found to stimulate apoptosis via external and internal pathways. Pycnidione also caused a decrease in the mitochondrial membrane potential and a significant increase in the level of reactive oxygen species and protein PAI-1 in A549 cells^14^. In Chinese medicine, the anti-cancer properties of compounds isolated from *Ganoderma* spp. against lung cancer are well known^15^.

Regarding preparations of animal origin, Arulvasu et al.^16^ showed that sardine (*Sardinella longiceps*) oil emulsion induced apoptosis and inhibited proliferation of A549 lung cancer cells in a dose- and incubation time-dependent manner. The probable mechanism of action of Ω-3 acids consists in interaction with the cell membrane and inhibition of membrane enzymes, which leads to apoptosis of neoplastic cells. Regarding invertebrates, it has been observed that proteins from the coelomic fluid of earthworms also show antitumor activity, for example against lung cancer^17^.

Earthworms are a source of anticancer substances. These annelids are used in Far East medicine, e.g., in Vietnam, China, or Korea, for treatment of many illnesses, e.g., burns, asthma, bronchitis, cardiovascular diseases, or gastrointestinal ulcers^17,18^. In their habitat, which has rich microbiota and ensures constant contact between microorganisms and earthworms, these animals have evolved mechanisms preventing development of illnesses^18–21^. The most popular formulations made from earthworms are powders and pastes^20–24^. It has been evidenced that coelomic fluid is rich in proteins, enzymes, peptides, and polysaccharides. It has fibrinolytic properties, exerts vasorelaxant effects, and has beneficial properties for the liver^17,18,22^. Earthworm preparations have documented antibacterial and antifungal properties^20,21,24–28^. Current research has demonstrated that coelomic fluid has anticancer activity against HeLa, A549, Pa17, PC-12, or Hep-2 cancer cell lines^20,26,29^. A mechanism of the coelomic fluid action against cancer cells based on limitation of glucose uptake by cells has been proposed^20^. Additionally, coelomic fluid has been shown to have no cytotoxic effects against human fibroblasts or erythrocytes^25,30^. These properties make earthworm-derived products promising agents in cancer therapy.

## Results

### MTT analysis

**Figure 1:**
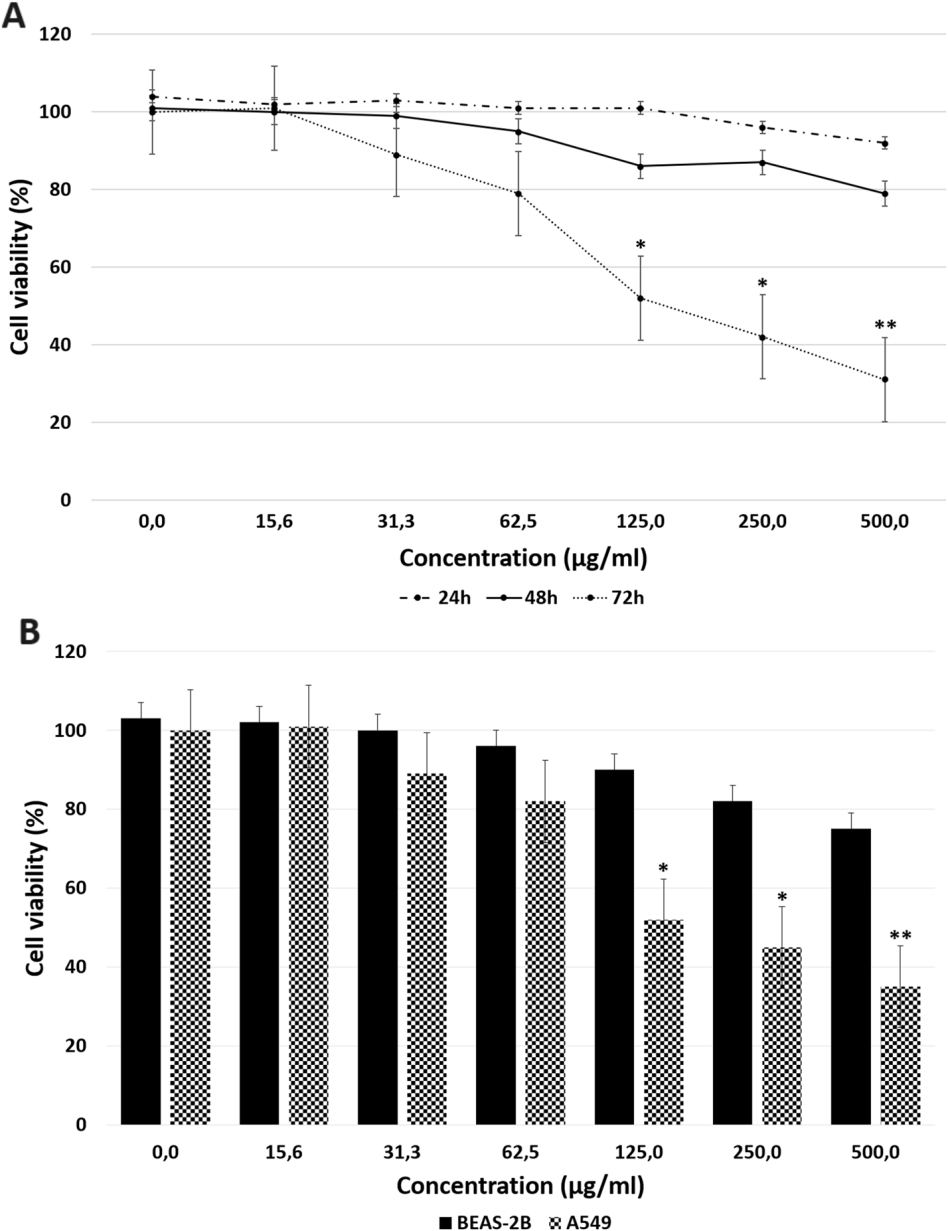
MTT assay results and effects of the indicated concentrations of Venetin-1. (**A**) Dose–response curves for Venetin-1 in A549 cells following 24-h, 48-h, and 72-h incubation. (**B**) Comparison of the cytotoxic activity of Venetin-1 against lung cancer A549 cells and normal BEAS-2B cells after 72-h incubation. The data are presented as means ± standard deviation (SD) from 3 independent experiments. *p < 0.05; **p < 0.01. Venetin-1 at the concentration in the range of 125–500 μg/mL after the 72-h incubation caused a significant decrease in the viability of the A549 cells, but induced no significant changes in the viability of the BEAS-2B cells.

The effects of Venetin-1 on the proliferation of the BEAS-2B and A549 cells were determined with the use of the MTT assay for 24, 48, and 72 h (Fig. 1). Although the A549 cell line was approximately 80–90% viable after the 24- and 48-h exposure to the different Venetin-1 concentrations (15.6–500 μg/mL), its viability after the 72-h incubation significantly declined to 52%, 41%, and 32% at the Venetin-1 concentrations of 125 μg/mL, 250 μg/mL, and 500 μg/mL, respectively (Fig. 1A). The viability of the BEAS-2B cells decreased to 75% in the 500 μg/mL Venetin-1-treated group at 72 h (Fig. 1B). At the lower concentrations, the BEAS-2B cells seemed to exhibit higher tolerance of Venetin-1, with greater viability (80–100%) at all the other Venetin-1 concentrations after the 72-h period.

### Venetin-1 provokes apoptosis activation in A549 cells

Apoptotic cell death induced by the Venetin-1 extract was investigated by monitoring phosphatidylserine (PS) translocation using the Annexin V-FITC/PI assay. Based on the ability of Annexin V to bind to PS, which is localized in the inner membrane leaflet of viable cells, the cells were classified as follows: LL—viable cells (Annexin V^−^/PI^−^), LR—early apoptotic cells (Annexin V^+^/PI^−^), UR—late apoptotic cells (Annexin V^+^/PI^+^), and UL—necrotic (Annexin V^−^/PI^+^) cells.

**Figure 2:**
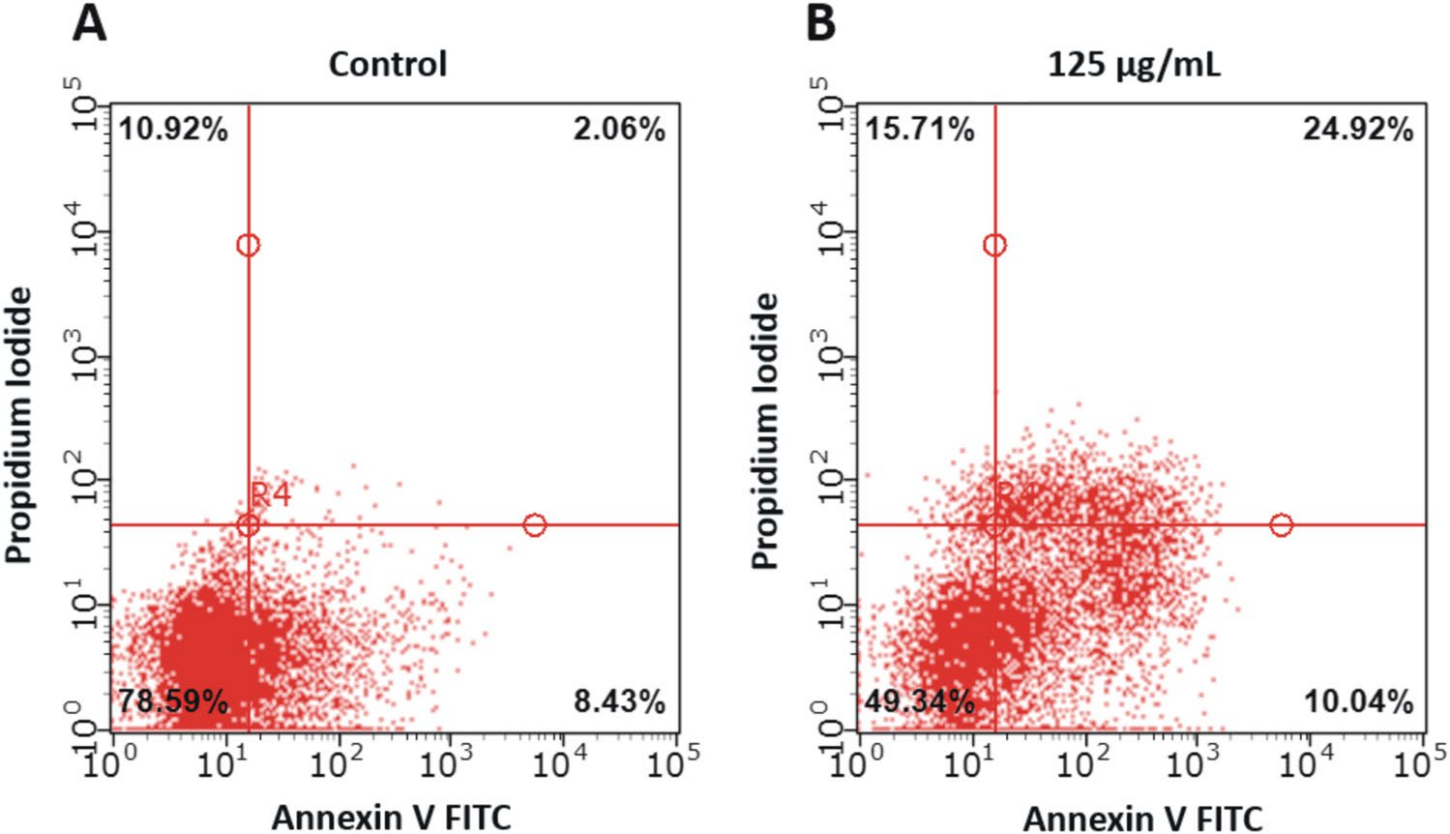
Apoptotic and necrotic activity of Venetin-1 in A549 cells assessed with the Annexin V/PI staining assay. Panel (**A**) shows representative dot plots of A549 control cells (without incubation with Venetin-1) after double staining with Annexin V-FITC and PI. Panel (**B**) shows the effects of incubation with Venetin-1 (125 µg/mL) for 72 h on cell apoptosis in A549 cells. The cells were classified as viable cells (lower left square), early apoptotic cells (lower right square), late apoptotic cells (upper right square), and necrotic cells (upper left square). Representative images of three independent experiments are shown. Venetin-1 had a necrotic and apoptotic effect on the A549 cells. The rate of necrosis as well as early and late apoptosis in the Venetin-1-treated A549 cells was higher by 5%, 3%, and 22.13%, respectively, than in the control.

We observed that the 72-h treatment with Venetin-1 had a necrotic and apoptotic impact on the A549 cells (Fig. 2). The number of viable cells dropped significantly from 77.03 ± 2.62% in the control to 48.41 ± 0.92% after the 72-h incubation with 125 µg/mL of Venetin-1. Compared to the control cells, an increased amount of necrotic (~ 5%) and early apoptotic cells (~ 3%) was observed in the Venetin-1-treated A549 cells. Furthermore, the cells exhibited a remarkably higher late apoptosis rate, compared to the untreated cells (24.31 ± 0.57 vs. 2.18 ± 0.27) (Table 1). The results of the activity of Venetin-1 against normal lung epithelial cells (BEAS-2B) are presented in the supplementary materials (Supplementary Fig. S1).

**Table 1:** Effects of 72-h incubation with 125 µg/mL of Venetin-1 on the cell type distribution in the A549 cell line. The results are expressed as mean ± SD values from three independent experiments. p-values lower than 0.05 (*p < 0.05, **p < 0.01), compared with the control group, are considered statistically significant.

	A549 control cells	Venetin-1-treated A549 cells	p-value
Viable cells (LL)	77.03 ± 2.62	48.41 ± 0.92	0.0001**
Necrotic cells (UL)	10.54 ± 0.73	15.60 ± 0.75	0.0011**
Late apoptotic cells (UR)	2.18 ± 0.27	24.31 ± 0.57	< 0.00001**
Early apoptotic cells (LR)	8.47 ± 0.04	11.68 ± 1.50	0.0211*

### Effect of Venetin-1 on the cell cycle analyzed with flow cytometry

**Figure 3:**
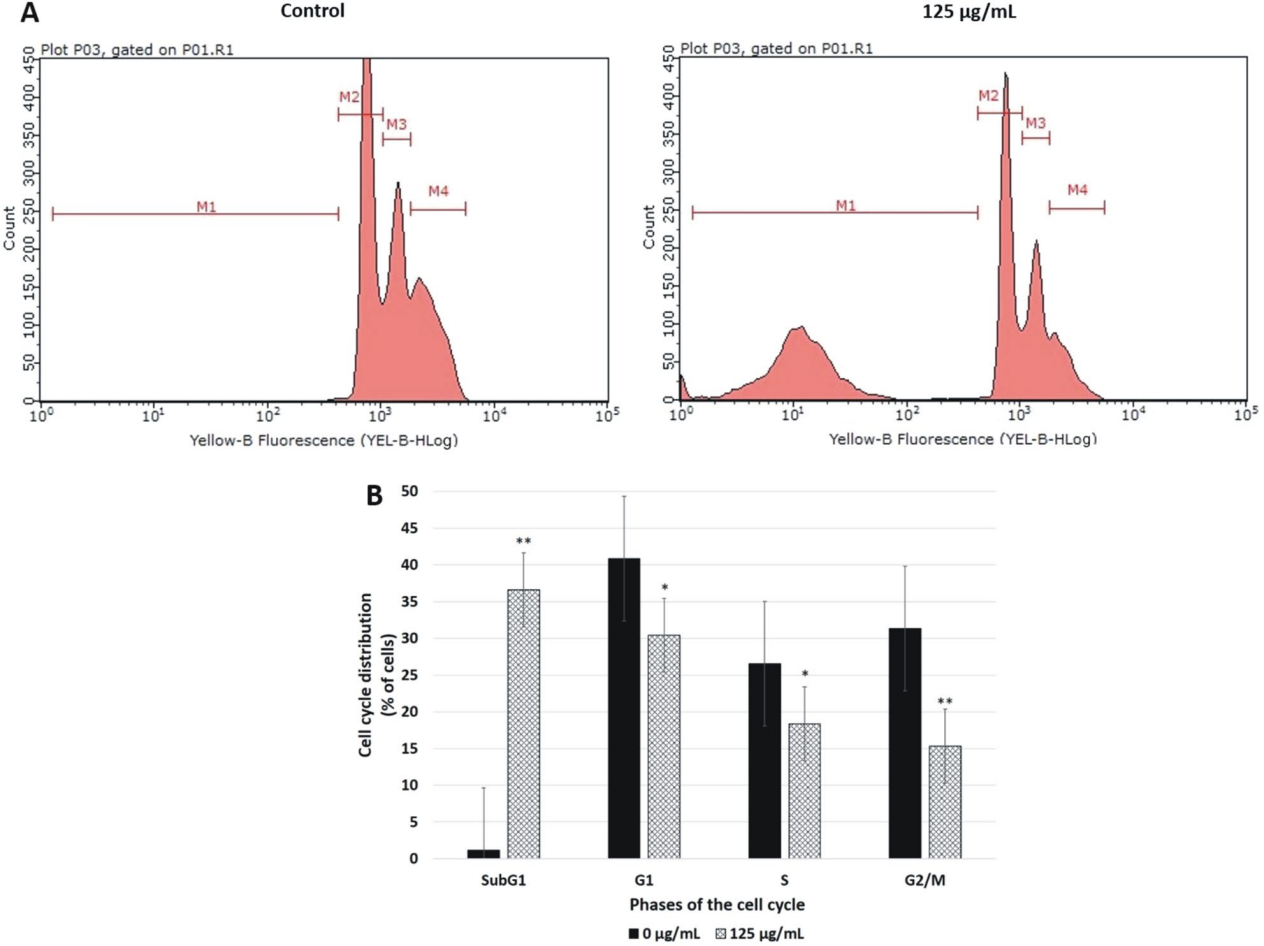
Effect of Venetin-1 treatment on A549 cell cycle phases. (**A**) Cell cycle analysis by flow cytometry in A549 control cells and those treated with Venetin-1 (125 µg/mL) for 72 h. The cell cycle distribution of PI-labeled cells was analyzed by flow cytometry analyses. The peaks in the illustration correspond to the sub-G1 (gate M1), G1 (gate M2), S (gate M3), and G2/M (gate M4) phases of the cell cycle. (**B**) Histogram showing the percentages of cells at each phase of the cell cycle. The results are expressed as mean ± SD values from three independent experiments. P-values lower than 0.05 (*p < 0.05, **p < 0.01), compared with the control group, are considered statistically significant. The Venetin-1 treatment caused arrest of A549 cells at the sub-G1 phase with an increase in the number of apoptotic cells and visible reduction in the proportions of treated and control A549 cells at the other cell cycle phases.

To examine the potential effect of the Venetin-1 treatment on the cell cycle, the A549 cells were treated with 125 µg/mL of Venetin-1 for 72 h, stained with PI, and subjected to flow cytometry analysis. As shown in Fig. 3, the treated A549 cells were arrested at the sub-G1 phase with a substantial increase (p < 0.0001) in the number of apoptotic cells (1.13 ± 0.08% vs. 36.63 ± 0.61%). The incubation with Venetin-1 also caused a concomitant decrease in the proportion of cells at the G1 (40.94 ± 0.94% vs. 30.44 ± 0.57%, p = 0.0001), S (25.56 ± 0.65% vs. 18.36 ± 0.50%, p = 0.0001), and G2/M (31.36 ± 0.35% vs. 15.32 ± 0.38%, p < 0.0001) phases of the cell cycle in the treated A549 cells, compared to the control.

### Determination of the concentration of caspases

**Figure 4:**
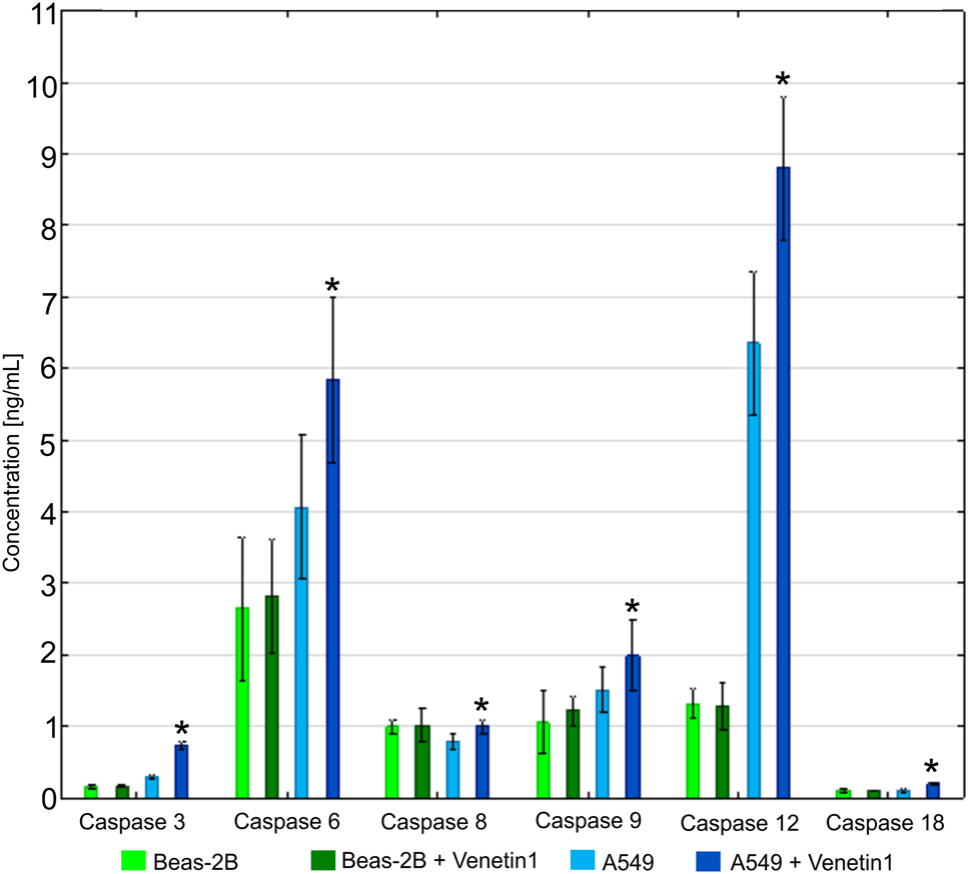
Concentrations of caspases 3, 6, 8, 9, 12, and 18 in BEAS-2B and A549 cell cultures. *p < 0.05. The biggest increase in the concentration of the tested caspases was observed in the culture of the A549 lung cancer cells treated with Venetin-1.

The level of caspases 3, 6, 8, 9, 12, and 18 was determined in homogenates of normal and neoplastic cells with and without Venetin-1 (Fig. 4). In the culture of the A549 lung cancer cells, the level of each of the tested caspases was higher in the cultures supplemented with Venetin-1 at the concentration of 125 µg/mL than in the untreated cultures. The level of caspases in the culture of the normal BEAS-2B cells with the addition of Venetin-1 at the concentration of 125 µg/mL did not differ significantly between the two studied groups.

After the incubation with Venetin-1 (125 µg/mL), the greatest increase in the concentration of caspases 3, 6, 8, and 9 was recorded in the culture of the A549 lung cancer cells. The highest increase in the caspase 12 concentration was recorded in the A549 lung cancer cell culture incubated with the active compound (Fig. 4). A slight decrease in the concentration of caspase 12 was noted in the culture of BEAS-2B cells with the addition of Venetin-1 (125 µg/mL). The greatest increase in the concentration of caspase 18 after the incubation with Venetin-1 was noted in the culture of the A549 lung cancer cells. A slight decrease in the concentration of caspase 18 was observed in the culture of normal cells incubated with Venetin-1. Full data are available in Supplementary materials (Supplementary Table S2).

### Light and fluorescence microscopy analysis of the Venetin-1 action on A549 cells

**Figure 5:**
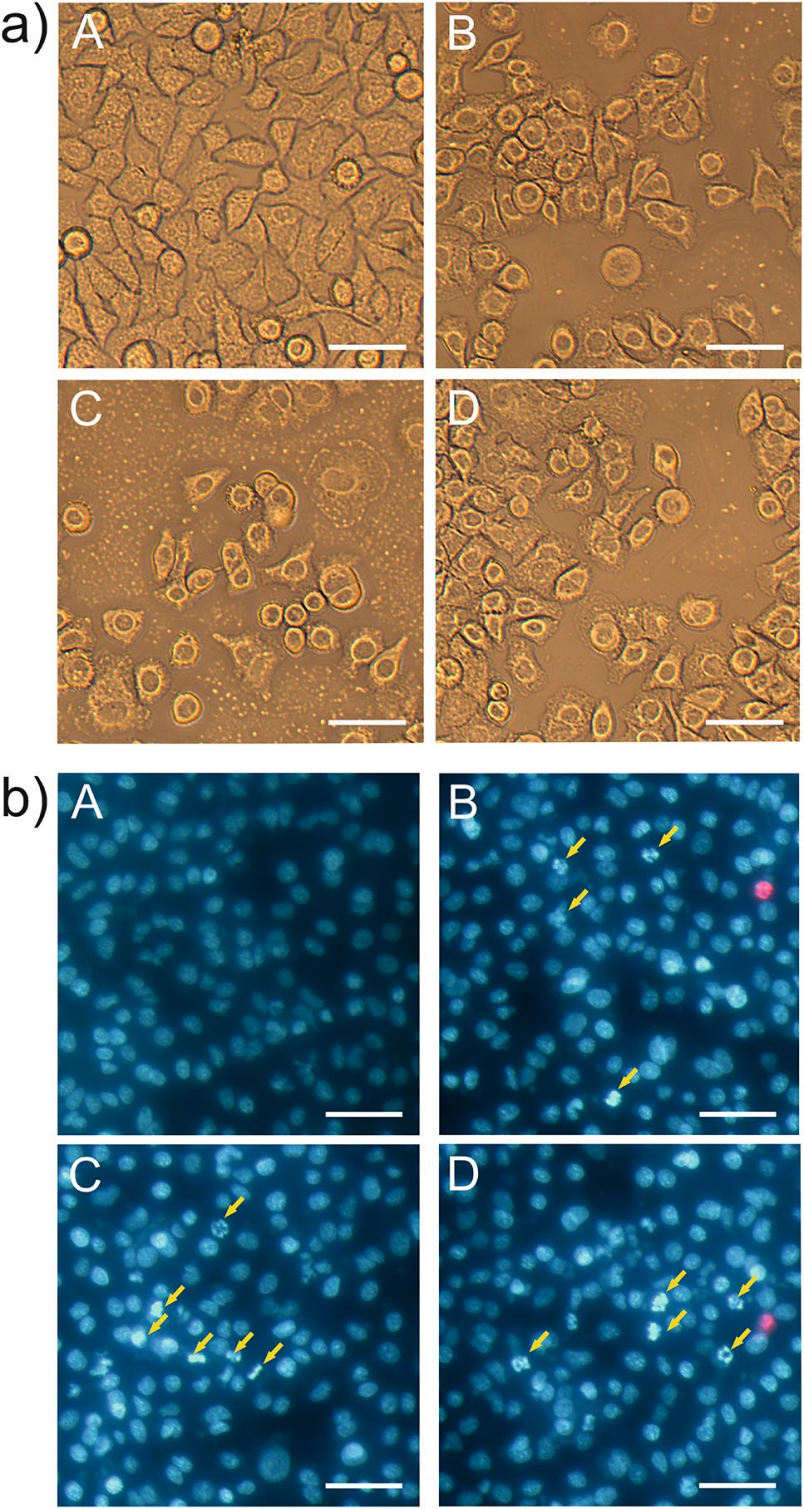
Visualization of Venetin-1-treated A549 cells with the use of transmitted light and fluorescence microscopy: (**a**) cytotoxic effect of Venetin-1 on A549. A—A549 control culture, B—A549 after 72-h incubation with Venetin-1 (62.5 µg/mL) at 78% cytotoxicity; C1, C2—A549 after 72-h incubation with Venetin-1 (125 µg/mL) at 48% cytotoxicity. The cells were visualized using a Zeiss Axiovert 40CFL microscope (Carl Zeiss). The scale bar corresponds to 20 µm. The images represent 10 images. The Venetin-1-treated cells were deformed and degraded; (**b**) apoptotic and necrotic effects on A549 cells after 72-h incubation with Venetin-1. A—control culture of A549, B—A549 cells treated with Venetin-1 (62.5 µg/mL), C1, C2—A549 cells treated with Venetin-1 (125 µg/mL). The yellow arrows indicate apoptotic cells with clear progressive degeneration of the nucleus. The pink cells are considered necrotic. The scale bars correspond to 50 µm. Each image represents 10 captured photos.

The analysis of the A549 control culture and variants incubated with 62.5 and 125 µg/mL of Venetin-1 showed that the cells treated with the active fraction were less clearly noticeable under the lens and were deformed. Cytopathic changes were frequently observed in the cells. In addition, strongly degraded or disintegrating cells were visible (Fig. 5a).

Cultures observed under the transmitted light microscope were also imaged using a confocal microscope after application of a mixture of fluorescent dyes to identify necrotic and apoptotic cells. The control cells were characterized by a regular dark fluorescent nucleus. Cells incubated with Venetin-1 at the concentrations of 62.5 and 125 µg/mL had bright luminous nuclei with frequently fragmented genetic material. Cells showing signs of apoptosis are marked in the image with arrows. Necrotic cells were sporadic and emitted red fluorescence (Fig. 5b).

### DIC and SEM visualization of A549 cells after Venetin-1 treatment

**Figure 6:**
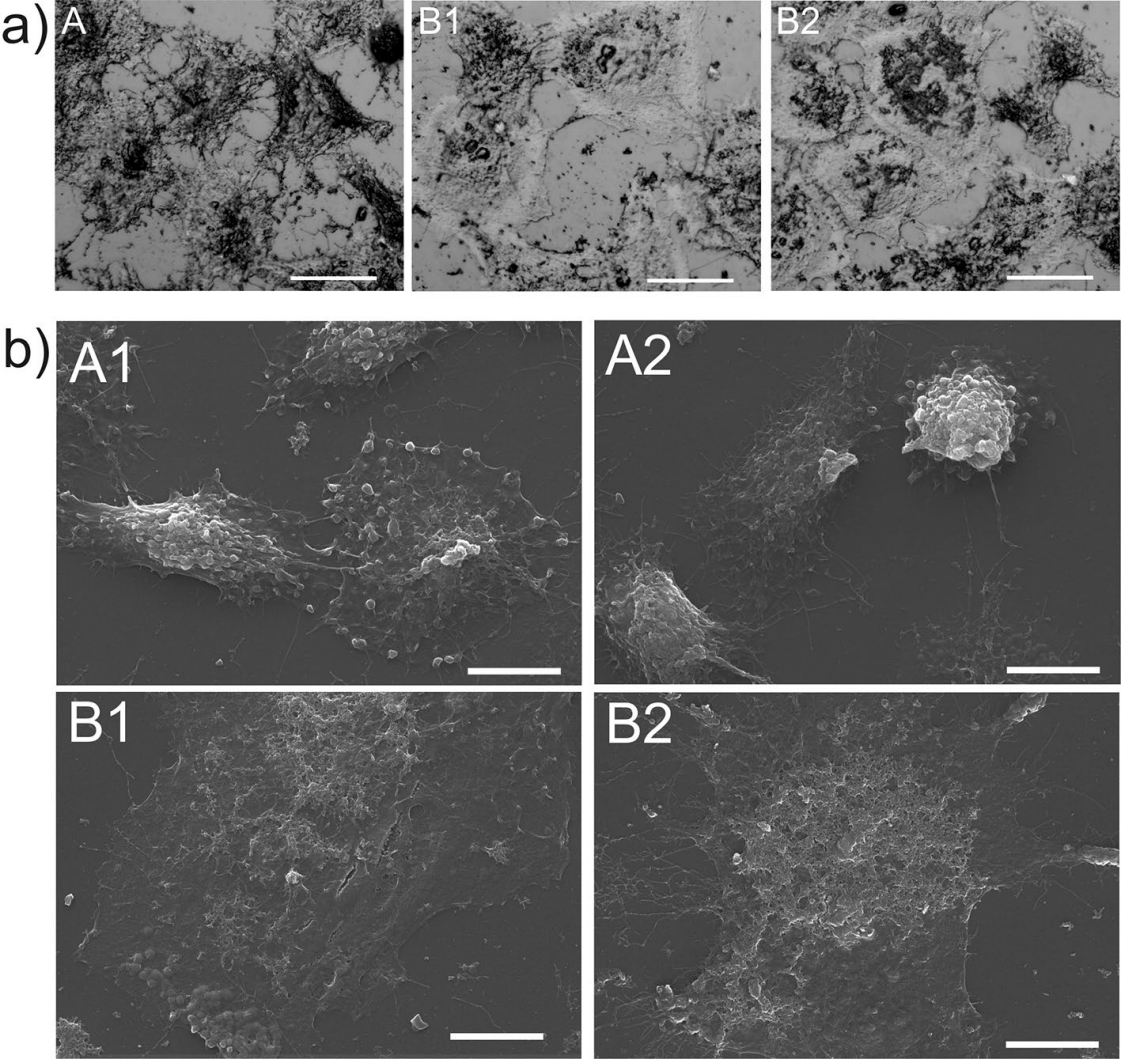
DIC and SEM analysis of A549 cells after exposure to Venetin-1: (**a**) DIC images of A549 cells after Venetin-1 treatment A—control culture of A549 B1, B2—A549 cells after incubation with Venetin-1 (125 µg/mL). The scale bars correspond to 20 µm. (**b**) SEM images of the surface of A549 cells after Venetin-1 treatment. A1, A2—control culture of A549, B1, B2—A549 cells after incubation with Venetin-1 (125 µg/mL). The scale bars correspond to 10 µm. After incubation with Venetin-1, contraction and degradation of cell structures were visible at the surface of the A549 cells. Each image represents 10 captured photos.

The control culture and cells incubated with Venetin-1 at the concentration of 125 µg/mL were observed using the DIC technique. The technique ensures the effect of a three-dimensional surface. It was observed that the control A549 cells were visibly denser and compact in the microscopic view, as indicated by their darker color and greater diversity of the observed surface (Fig. 6a A). After the Venetin-1-treatment, the cells contracted, which was evidenced by the concentrated dark pigmentation on the cell surface closer to their center (Fig. 6a B1,B2). The scanning electron microscopy of the tumor cells facilitated comparison of their surfaces between the control and Venetin-1 incubation (125 µg/mL) groups. The surface of the control cells was rich in fine granular structures (Fig. 6a A1,A2), whereas the Venetin-1-treated cells did not have these granules and their cell surface exhibited remnants of damaged structures (Fig. 6b B1, B2). The cell contraction effect was also visible as discernible cracks in the microscopic image (Fig. 6b B1).

### Atomic Force Microscopy (AFM) analysis of A547 cells

**Figure 7:**
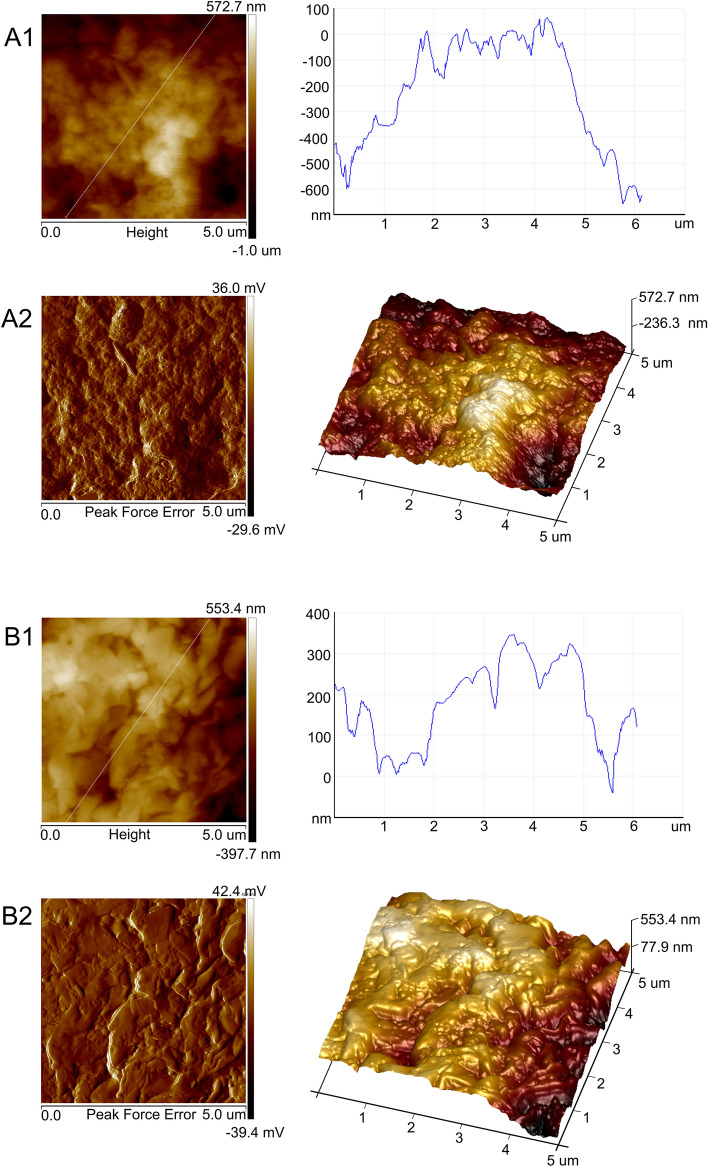
AFM analysis of the A549 cell wall surface. (**A1**, **A2**) Control culture of A549, (**B1**, **B2**) Cancer cells incubated with 125 µg/mL Venetin-1. Image of high altitude and peak force error—left panel; height profile and 3D image—right panel. Venetin-1 treatment caused changes in the cell wall topography and mechanical properties of the A549 cells, and significantly lower elasticity expressed as Young’s modulus was observed.

The topographic and peak force error images captured by AFM on the surface of the A549 cells incubated with 125 µg/mL of Venetin-1 revealed differences (Fig. 7B1,B2) from the control cells (Fig. 7A1,A2). The mean amplitude of the A549 control cells subjected to the height measurement was two-fold lower than the mean amplitude of the Venetin-1-treated cells. The A549 cells incubated with Venetin-1 had a markedly varied surface with visible indentations in the height profile (Fig. 7B1,B2). In order to determine cell surface areas with the lowest and the greatest elasticity, a Young’s modulus map was made and the Young’s modulus values for the given areas were compared. The average value of Young’s modulus of areas with the greatest elasticity was 2120.7 MPa in the control and 1142.8 MPa in the Venetin-1 treated A549 cells. Young’s modulus of areas with the lowest elasticity was 2.3 times lower (2647.4 MPa) in the A549 cells incubated with Venetin-1 than in the control cells (6300.9 MPa). In both cases, the differences were statistically significant (p < 0.001; Student’s T-Test). The AFM images indicated apoptotic morphological and biophysical changes in the A549 cells caused by the Venetin-1 treatment.

### Proteomic analysis

Before the enzymatic digestion of the protein components of Venetin-1 and DVr (raw coelomic fluid), the samples were reduced with DTT and subjected to electrophoretic separation in the SageELF automatic system. Proteins were separated in the size-based mode in the 10–300 kDa range. After separation, the system automatically eluted the proteins from the gel into separate wells in the cassette, from which they were pipetted into Eppendorf tubes and digested according to the FASP protocol (each fraction separately). The protein identification results for Venetin-1 in each fraction are presented in Table 2 (all results are available in the Supplementary materials, Table S3 for Venetin-1 and S4 for raw coelomic fluid DVr). Identification was carried out based on the revised protein database for Annelida (Uniprot).

**Table 2:** List of proteins identified in the Venetin-1 preparation after separation in the 3% SDS-Agarose cassette (full table available in Supplementary materials, Table S3).

Description	Accession	Coverage (%) Fr1_T	Coverage (%) Fr2_T	Coverage (%) Fr3_T	Coverage (%) Fr4_T	Coverage (%) FR5_T	Coverage (%) Fr6_T	Coverage (%) FR7_T	Coverage (%) FR8_T	Coverage (%) FR_9_T	Coverage (%) Fr10_T	Coverage (%) Fr11_T	Coverage (%) Fr12_T	Avg. mass
**Lysenin**	**O18423**	**51**	**40**	**48**	**33**	**12**	**12**	**23**	**27**	**76**	**66**	**23**	**12**	**33,441**
**Actin-2**	**P92176**	**36**	**37**	**50**	**47**	**23**	**19**	**29**	**45**	**58**	**42**	**15**	**8**	**41,840**
**Lysenin-related protein 2**	**O18425**	**49**	**39**	**49**	**38**	**23**	**23**	**28**	**32**	**73**	**64**	**24**	**18**	**34,143**
Gelsolin-like protein 1	Q7JQD3	3	18	25	11	11	0	10	17	31	14	3	0	41,636
Lysenin-related protein 3	Q3LX99	9	6	6	6	6	6	6	6	21	6	6	6	33,841
Lysenin-related protein 1	O18424	6	6	6	9	3	3	3	6	11	6	0	0	33,913
Calmodulin	Q9GRJ1	0	0	0	0	0	0	0	0	9	27	40	22	16,841
Chymotrypsin inhibitor (Fragment)	P83830	22	22	22	22	22	22	22	22	22	22	24	24	4501
Extracellular globin-4	P13579	0	0	0	0	0	0	0	0	9	20	23	20	17,525
Extracellular globin-2	P02218	0	0	0	0	0	0	0	0	6	0	28	29	16,254
Extracellular globin-3	P11069	0	0	0	0	0	0	0	0	6	0	15	13	19,082
Histone H4	Q7KQD1	0	0	0	0	0	0	0	0	0	0	17	29	11,367
Histone H4	P62795	0	0	0	0	0	0	0	0	0	0	17	29	11,367
Histone H4	P62794	0	0	0	0	0	0	0	0	0	0	17	29	11,367
Ubiquitin (Fragment)	P84589	40	40	40	40	0	0	14	0	40	40	40	40	7203
Translationally controlled tumor protein homolog	O18477	0	0	0	0	0	0	0	0	0	0	21	9	18,837
Sarcoplasmic calcium-binding protein	P04572	0	0	0	0	0	0	0	0	0	0	6	6	19,525
Sarcoplasmic calcium-binding protein	P04571	0	0	0	0	0	0	0	0	0	0	6	6	19,486
Neurohemerythrin	Q674M7	0	0	0	0	0	0	0	0	0	0	0	9	13,814
Histone H2A	P19178	0	0	0	0	0	0	0	0	0	0	7	7	13,310
Histone H2A	P27325	0	0	0	0	0	0	0	0	0	0	7	7	13,440
40S ribosomal protein S13	O77303	0	0	0	0	0	0	0	0	0	0	7	0	17,115
60S ribosomal protein L4	P49165	0	0	0	0	0	0	0	0	3	0	0	0	43,135
Catenin beta	P35224	0	0	0	0	0	0	0	0	0	0	2	0	89,070
Chymotrypsin inhibitor	P83472	0	0	0	0	0	0	0	0	0	0	0	17	9366
Fibril-forming collagen alpha chain (Fragment)	P30754	0	0	0	0	0	0	0	0	0	0	1	0	94,587

Significant values are in [bold].

**Figure 8:**
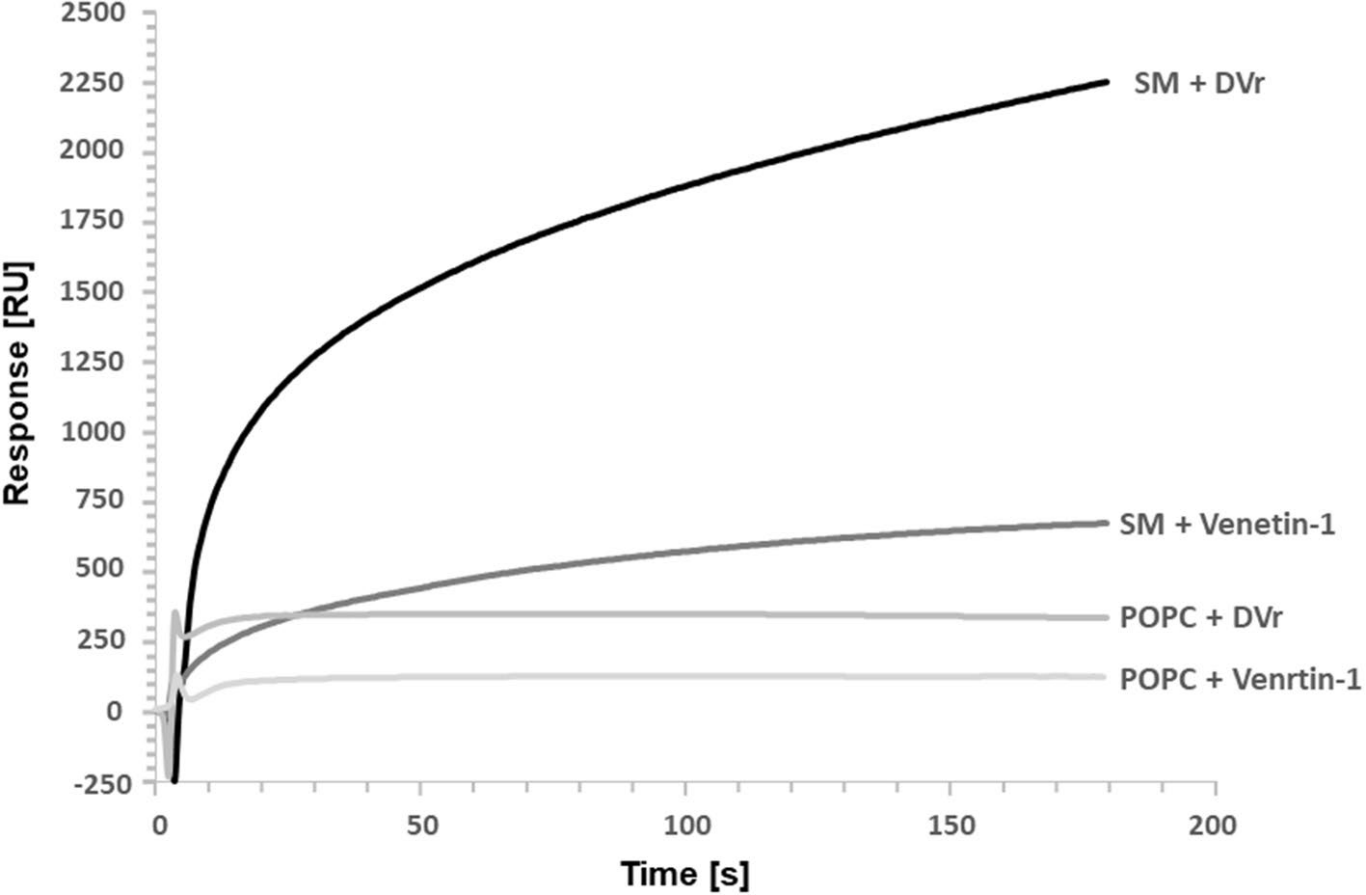
SPR analysis of protein extracts (DVr—raw coelomic fluid and Venetin-1) binding to SM (sphingomyelin) and POPC (1-palmitoyl-2-deoylphpsphatydilcholine). The observed changes were related to the reduction in Venetin-1 binding to sphingomyelin in comparison to the unprocessed DVr preparation, the lower binding of DVr to the POPC lipid, and the weaker binding of Venetin-1 to POPC.

The binding of the Venetin-1 preparation and its precursor—DVr (raw coelomic fluid) collected from earthworms and unprocessed (without heating and dialysis) to two lipids: sphingomyelin and POPC was determined. 3-mM liposome solutions were prepared according to the procedure described in the Materials and Methods section. The lipids were deposited on the L1 sensor designed to study the interaction of proteins with lipids. Then, after blocking the sensor with an albumin solution, the procedure of binding of both preparations was performed, and the bound proteins were eluted and subjected to proteomic analysis. The sensorgrams presented in Fig. 8 show the binding of the preparations to lipids. There was a significant reduction in Venetin-1 binding to sphingomyelin compared to the unprocessed DVr preparation. Lower binding of DVr to the POPC lipid was detected. The Venetin-1 binding to POPC was weaker and was associated with quick detachment from the complex.

The proteomic analysis of the recovery fractions from the SPR experiments showed that only one protein was identified in each case, namely lysenin-related protein 2 (LRP2). The highest sequence coverage and most peptides were identified in DVr. For details, see Table S5 in the Supplementary materials.

### SEM and Cryo-TEM analysis of Venetin-1

**Figure 9:**
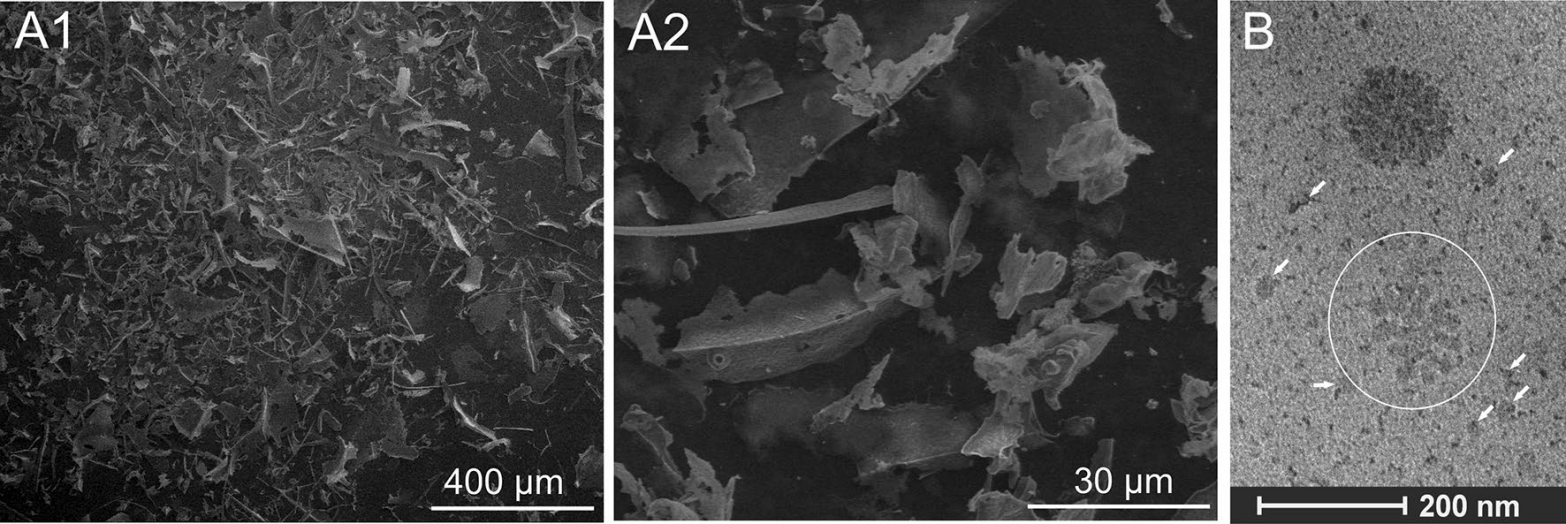
Imaging of Venetin-1: (**A1**, **A2**) SEM images of Venetin-1; (**B**) Cryo-TEM imaging of Venetin-1. The image shows small structures grouped into a larger one (marked with a white circle). Loose structures are marked with arrows. A dark circular compact structure formed of smaller particles is visible in the upper part of the image.

The active compound Venetin-1 was visible under a scanning microscope as small flakes of various sizes. The longest structures were about 200 µm in length (Fig. 9A1,A2). The structure of the preparation was also observed using the Cryo-TEM technique. It was noticed that the smaller structures clustered together to form a larger circular structure. The accumulation of the smaller structures is indicated by the white circle in Fig. 9B, while the loose structures are indicated by arrows. A compact, round shape is visible in the upper part of the specimen. The observed image suggests that the Venetin-1 structure is characteristic of a polymeric compound.

### XPS analysis of Venetin-1

The analysis showed the presence of carbon (64.1%), oxygen (23.5%), nitrogen (6.3%), phosphorus (1.6%), and sulfur (0.3%). All these elements are characteristic of proteins. The tests also showed the presence of chlorine (3.0%) and sodium (1.3%).

**Figure 10:**
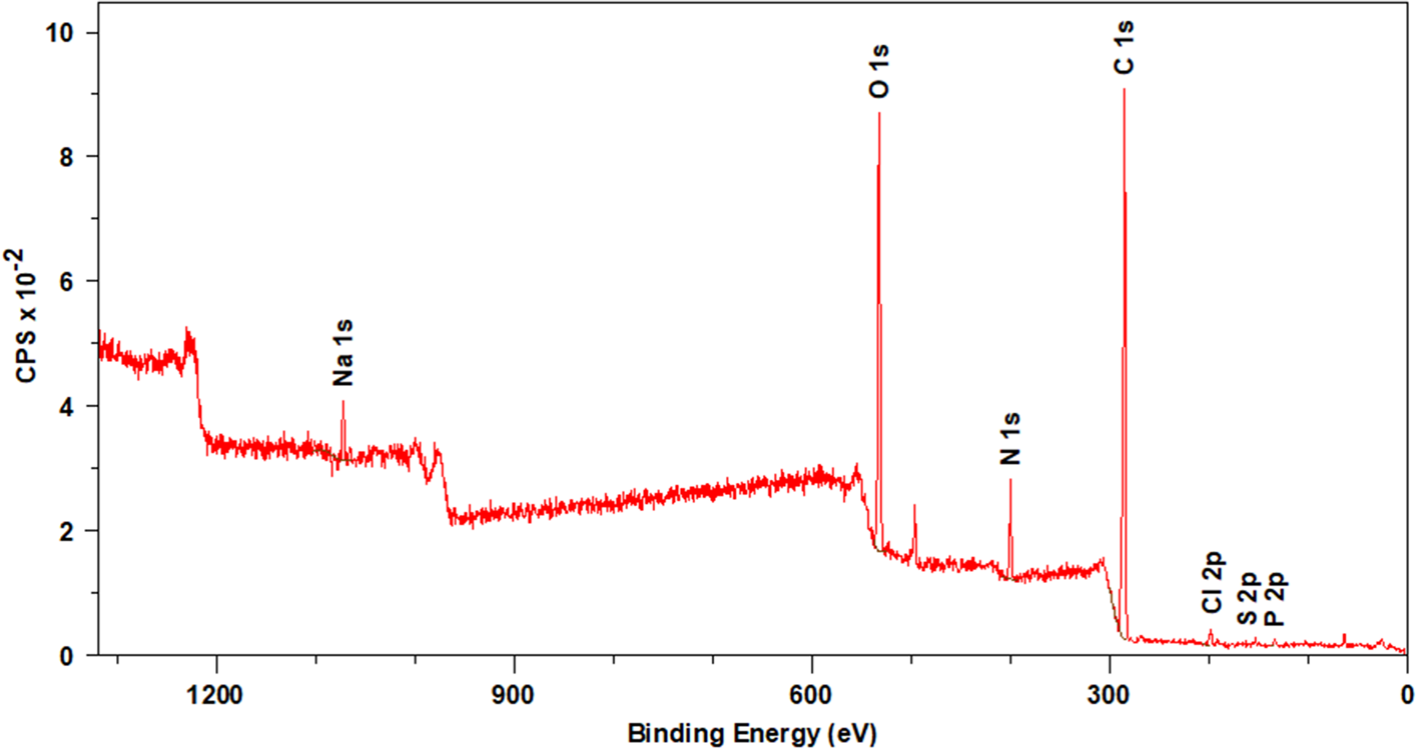
XPS survey spectrum of Venetin-1. Elements characteristic of proteins were detected, including: carbon, nitrogen, phosphorus, and sulfur; additionally, the presence of chlorine and sodium was demonstrated.

The XPS survey spectrum of Venetin-1 is shown in Fig. 10. The quantitative results of the XPS analysis of the elemental composition of the Venetin-1 surface are presented in Table 3.

**Table 3:** Quantitative results of the XPS analysis of the elemental composition of the Venetin-1 surface.

Name	Position (eV)	Raw Area	%At Conc	% Mass Conc
C 1s	285.0	2522.490	71.6	64.1
N 1s	400.0	384.404	6.1	6.3
O 1s	532.0	2036.990	19.7	23.5
Na 1s	1072.0	223.642	0.7	1.3
P 2p	133.5	28.229	0.7	1.6
S 2p	162.5	6.283	0.1	0.3
Cl 2p	197.5	89.987	1.1	3.0

**Figure 11:**
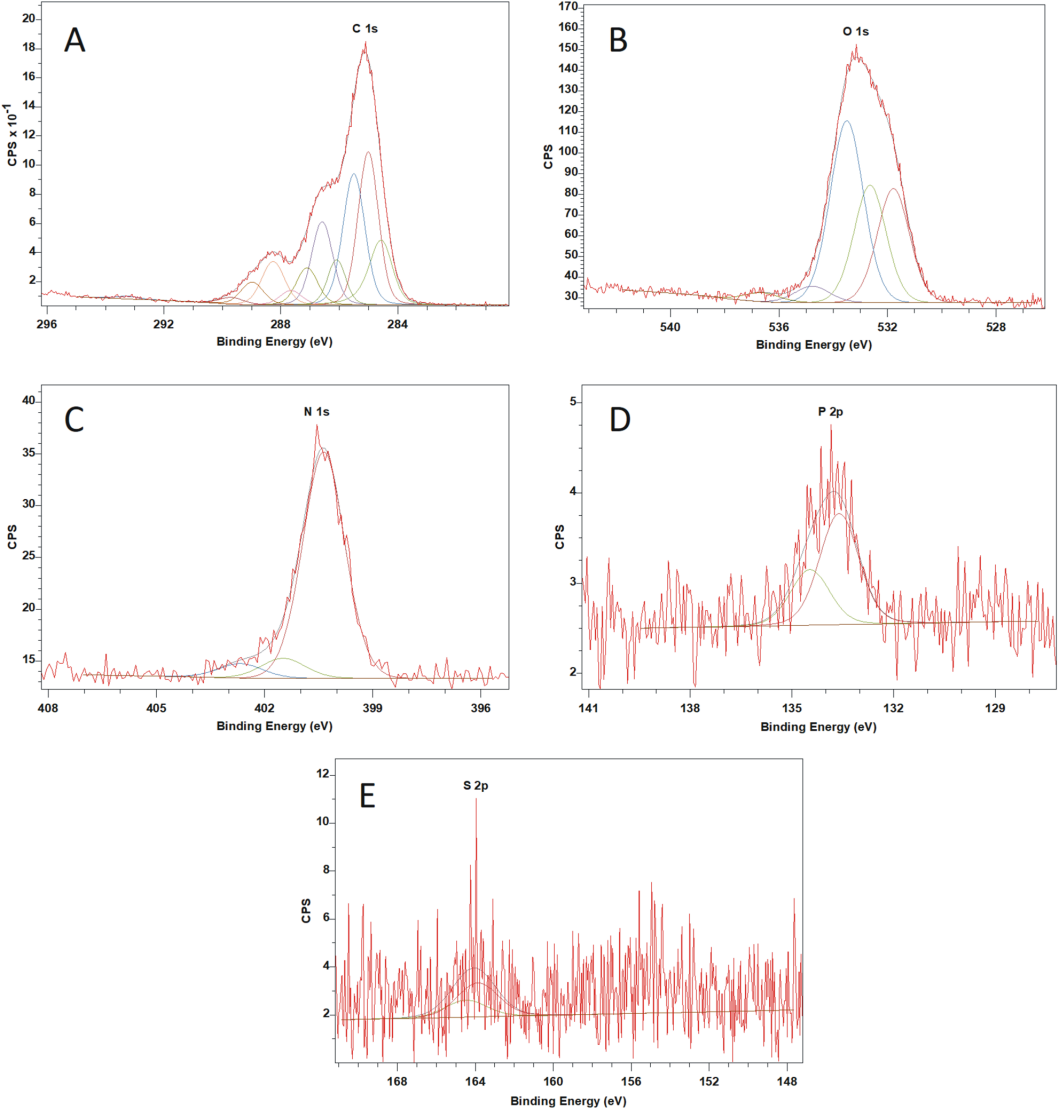
XPS spectra of Venetin-1 in a narrow range of binding energies for: (**a**) carbon, (**b**) oxygen, (**c**) nitrogen, (**d**) phosphorus, (**e**) sulfur.

To identify chemical bonds present in the analyzed preparation, spectra in a narrow range of binding energies for carbon, oxygen, nitrogen, sulfur, and phosphorus were obtained. After a pick fitting operation based on the literature data, the chemical bonds were adjusted for the individual binding energies. Figure 11 shows the spectra in a narrow range of XPS binding energy. Table 4 presents the elemental composition of Venetin-1 with identification of characteristic chemical bonds.

**Table 4:** Elemental composition of Venetin-1 with identification of characteristic chemical bonds^31–36^.

Name	Position (eV)	Raw area	%At Conc	Species
C 1s A	285.00	95.275	24.2	C–C sp3
C 1s B	284.53	48.347	12.3	C=C sp2
C 1s C	285.51	88.619	22.5	C–H
C 1s D	286.59	53.979	13.7	C–OH
C 1s E	287.11	24.327	6.2	C–O–C
C 1s F	287.66	9.004	2.3	C=O
C 1s G	288.26	30.138	7.6	O=C–O–
C 1s H	288.98	15.387	3.9	O=C–OH
C 1s I	289.70	4.988	1.3	CO_3_^–2^
C 1s C-N	286.10	24.342	6.2	C–N
N 1s A	400.36	32.361	86.1	–NH–
N 1s B	401.49	3.031	8.1	–NH^+^–
N 1s C	402.67	2.202	5.9	=NH^+^–
O 1s A	531.78	83.346	26.1	O=C–O
O 1s B	532.65	85.671	26.8	C–OH
O 1s C	533.50	137.338	43.0	C–O–C
O 1s D	534.78	12.883	4.0	O–C=O
P 2p 3/2	133.61	1.85823	50.53	P=O, PO_4_^3−^
P 2p 1/2	134.47	0.929117	49.47	
S 2p 3/2	163.87	3.91327	50.53	C–S–C
S 2p 1/2	164.48	1.95664	49.47	

The study showed the binding characteristics of both polysaccharides and proteins. The spectra in the narrow range of binding energies for carbon showed the presence of C–C, C–H, C–OH, C=O, and C–O–C bonds in the polysaccharides. The presence of the O=C–OH carboxylic bond characteristic for proteins was also observed. The C=O and C–N groups characteristic for the peptide bond were found as well. The tests performed in the narrow binding energy range characteristic of nitrogen showed the presence of nitrogen in the form of amine bonds. These bonds are characteristic of proteins. The presence of the organic form of phosphorus and sulfur also confirms the presence of proteins in the tested preparation.

### Dynamic light scattering domain-specific characterization of Venetin-1

**Figure 12:**
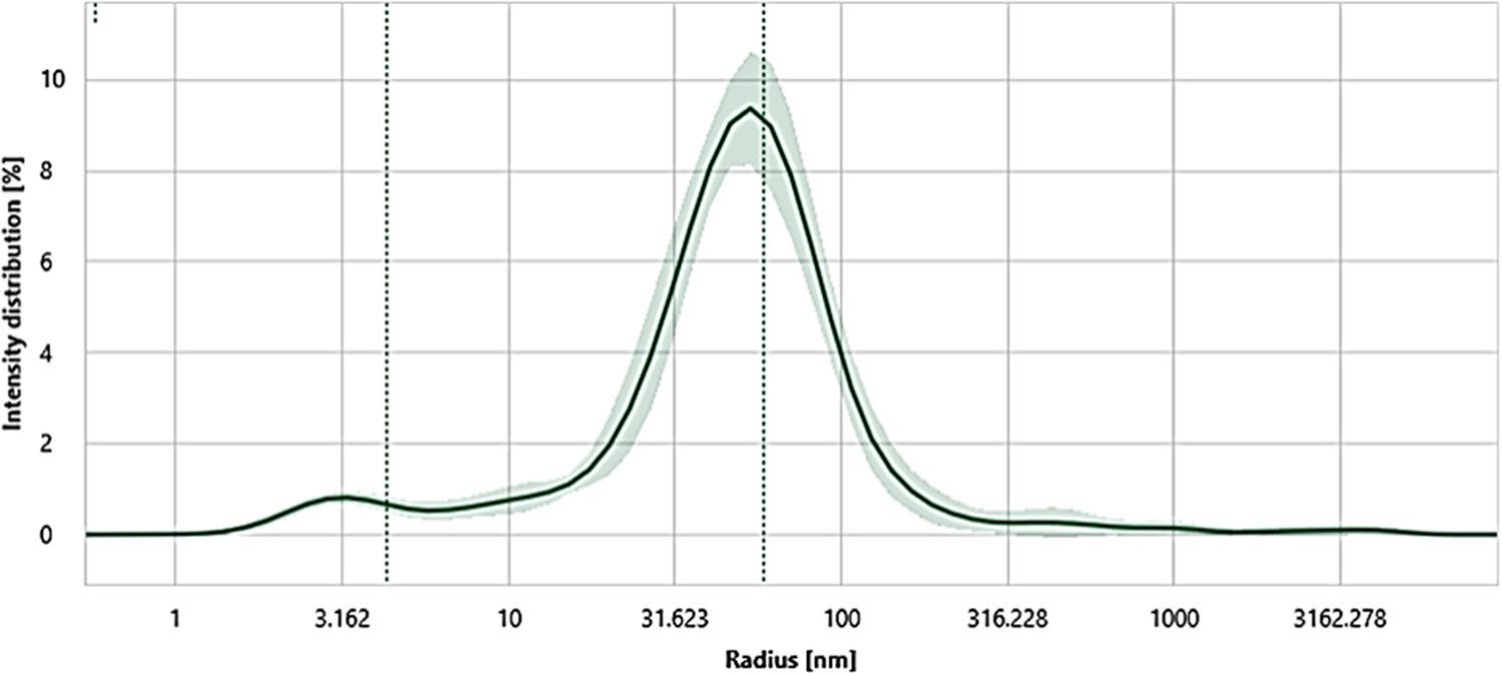
Prometheus Panta DLS size analysis of Venetin-1 microparticles. The preparation proved to be homogeneous in the size profile.

As seen in Fig. 12 and Table 5, the samples display a very homogeneous size analysis profile with the microparticle size determined to be 58.23 ± 3.93 nm.

**Table 5:** Prometheus Panta size analysis parameters.

Annotations	Intensity distribution				Acquisition	
	Peak 1 (nm)		PDI 1		Scattering Intensity (cts/s)	
Sample ID	ø	σ	ø	σ	ø	σ
Venetin-1	58.23	3.93	0.76	0.33	4,525,630.189	1,089,085.296

Following the size analysis conducted with Prometheus Panta, was performed a melting temperature experiment with a temperature gradient from 25 to 95 °C and a heating ramp of 1 °C/min (Table 6). As shown in Fig. 12, the microparticles displayed single 350/330 nm transition at a temperature of 64.95 ± 0.08 °C. This transition very probably corresponded to the melting point of the analyzed microparticles. Moreover, it was determined that, after the conformational change represented by the fluorescence ratio 350/330 nm, the analyzed microparticles underwent aggregation with the Tagg 66.92 ± 0.16 °C and aggregation onset 58.97 ± 1.15 °C.

**Table 6:** Prometheus Panta melting profile parameters.

Annotations	Ratio ↑				Turbidity ↑					
	IP #1 (°C)		ON (°C)		IP #1 (°C)		ON (°C)		Turbidity average	
Sample ID	ø	σ	ø	σ	ø	σ	ø	σ	ø	σ
Venetin-1	64.95	0.08	25.50	0.66	66.92	0.16	58.19	1.57	9.0	0.3

## Discussion

Renewable sources of medicinal products of animal origin, pharmaceutical preparations based on these sources, and biological food additives seem to be an integral part of the progress in the search for effective therapeutic agents. This kind of human activity should be improved in every possible way. Convenient animal models contribute to the development of complementary and alternative medicine called integrative medicine. Such invertebrates as earthworms are inexpensive, ethically non-controversial, and useful in understanding mechanisms underlying biological processes^37^. The use of pharmaceuticals derived from earthworms is highly developed as green medicine in China and other Asian countries. Extracts prepared from these invertebrates are used to treat many diseases. Among invertebrates, earthworms are a known source of antibacterial, antifungal, and anticancer compounds. The coelomic fluid of the earthworm body cavity exhibits many types of biological properties, e.g., antimicrobial, proteolytic, hemolytic, hemagglutinating, antifungal, and antitumor activity.

Our research shows that Venetin-1 characterized previously as the protein-polysaccharide fraction from the coelomic fluid of *D. veneta* earthworm has a selective effect on A549 lung carcinoma cells, destroying cancer cells and saving normal ones. The preparation was obtained through several processes, such as extraction of the fluid by means of electric shock, centrifugation, filtering, incubation, and lyophilization. A particularly important step was to deprive the fluid from cytotoxicity to normal cells while maintaining its high activity against tumor cells. These processes were described in the publication describing the action of fractions from coelomic fluid on A549 lung cancer cells^29^.

The preparation obtained caused apoptotic cell death. From the point of view of modern oncology, apoptosis is a very important process to be activated in cancer cells. Cancer cells are immortal in nature. Modern drugs are attempted to be designed to induce apoptosis (or some other type of cell death) in only abnormal cells, leaving healthy cells in the body intact. In other words, the tumor is pharmacologically induced to commit suicide. The induction of the apoptosis process is one of the main goals of the designed anti-cancer therapies. This is related to the developed mechanisms of tumor cell resistance to programmed death. Current chemotherapeutic drugs kill cancer cells mainly through induction of apoptosis. However, they become ineffective when the neoplastic cell can metastasize, which is associated with poor prognosis and high mortality^38^. Cancer cells usually have several gene mutations that affect the apoptotic process, thereby avoiding programmed cell death. This resistance to apoptosis is beneficial to metastatic cells^38–42^.

Target proteins are proteolyzed by caspases, which may lead to their activation or inhibition, thereby exerting a significant impact on the future fate of the cell. They perform many important functions in the organism. Above all, these proteins play a key role in apoptosis. In addition, they are involved in other cell death mechanisms such as pyroptosis, necrosis, and autophagy. They are also important in the functioning of the immune system and in inflammatory processes by stimulation of the secretion of pro-inflammatory cytokines. They also participate in the aging processes. Some caspases have anti-cancer properties^43–47^. Initiator caspases (caspases 2, 8, 9, and 10) are involved in the initial phase of apoptosis, and their role is to enhance the death signal. They are also responsible for the activation of effector caspases (caspases 3, 6, 7). In turn, after activation, executor caspases rapidly break down cellular elements^43,47,48^.

The present results indicate that Venetin-1-causes a statistically significant increase in caspase activity in cultured A549 lung cancer cells. The increase in the concentration of caspase 3, which belongs to the group of executor caspases, and the decrease in the number of living cells in the culture may indicate intensification of the apoptosis process in these tumor cells after the addition of Venetin-1. However, the increase in caspase 12 suggests that this preparation may also be important in the induction of the inflammatory process. The increase in the concentration of caspase 6 indicates that Venetin-1 influences the execution phase of apoptosis, and the increase in the concentration of caspases 8 and 9 reflects their influence on the initiation phase. In turn, no significant changes in the parameters tested were found after the addition of Venetin-1 in the culture of normal cells of the bronchial epithelium of the BEAS-2B bronchial tree.

The light microscopy, scanning electron microscopy, and atomic force microscopy observations revealed changes in the morphology and nanomechanical properties of the tumor cells after the incubation with Venetin-1. The microscopic images provided by the different techniques complemented each other. Transmitted light microscopy showed a decrease in the cell density and deformation after the incubation with the active compound. Changes in the A549 cell surface parameters, such as roughness, were detected by atomic force microscopy. Early apoptosis is characterized by remodeling of the cytoskeleton and nucleus. It induces dynamic changes in the nanomechanical properties of the cell surface^49^. Macroscopically, the reduction in culture density was also evidenced by the DIC technique.

The isolated Venetin-1 caused a significant increase in the level of caspases 3, 12, and 18 in the A549 lung cancer cells and at the same time induced changes in the cell viability and density in these cultures. This indicates that this preparation exerts its antitumor effect by stimulating the apoptosis process in A549 lung cancer cells.

Although we describe our preparation as a protein-polysaccharide fraction, it is in fact a complex, as previous electrophoretic analyses thereof showed that the signals coming from proteins were located exactly in the same positions as the signals coming from sugars. This indicates that these protein and sugar compounds are related to each other. In addition, FTIR spectroscopy analyses proved the chemical homogeneity of the preparation, since the FTIR spectrum looked the same in each analyzed region and had the same intensity. Another observation supporting the nature of the complex is the Cryo-TEM image of the preparation, which clearly showed identical smaller structures merging into a larger spherical form. The XPS analyses of the chemical bonds confirm this theory. Additionally, the Dynamic Light Scattering domain-specific analyses carried out with the use of Prometheus Panta showed that Venetin-1, previously regarded as a protein-polysaccharide fraction, was one nanoparticle.

Previous chemical analyses carried out in studies on antifungal activity^27,28,30^ showed that proteins of the lysenin family: lysenin-related protein 2 (LRP2) and lysenin, which were identified with an accuracy of 95% and 90% sequence coverage, respectively, were the main components of Venetin-1. Lysenin is a 33 kDa protein found in the coelomic fluid of earthworms^50^. Probably, lysenin is produced by coelomocytes and free chloragocytes, since lysenin mRNA has been found in these cells. Lysenin binds specifically to sphingomyelin (SM) and, when bound to the cell membranes of target cells, it forms oligomers in an MS-dependent manner. These proteins belong to the group of pore-forming toxins, which may be responsible for the biological activity of the preparation.

The SageELF (Electrophoretic Lateral Fractionation) system was used for separation of the protein components of DVr and Venetin-1. It is used for protein fractionation in an SDS agarose gel matrix with simultaneous elution. The software controls the fractionation time based on the signal from the fluorescent marker added during the sample preparation steps. In this way, the obtained fractions were divided according to the molecular size from 10 to 300 kDa. The proteomic analysis confirmed the presence of significant amounts of constituents in all eluates. In the case of Venetin-1, the largest share in fraction 9 (eluted from 8 to 10) is the monomeric form of lysenin, actin 2, and lysenin-related protein 2. However, it is not the only form present in the whole preparation. A high frequency of this form estimated by the PeaksStudio program parameters (Supplementary Table S3) was observed in higher molecular-weight fractions, from which molecules with the weight of about 260 kDa (fraction 1) to 100 kDa (fraction 4) were eluted. Assuming the mass of the monomer as the mass of lysenin or LRP2 (33/34 kDa), these may be trimer to octamer forms if they are homogeneous lysenin or heterogeneous LRP2 oligomers as a mixture of lysenin and LRP2. Their sequences are very similar (identity 89%). Due to the presence of actin 2 (42 kDa) and e.g. ubiquitin fragments identified in all fractions, it is also assumed that it may be a multiprotein complex. The sensorgram analysis revealed differences between Venetin-1 and DVr in the interaction with lipids. The data for DVr are consistent with literature reports pointing to lysenins as proteins that specifically recognize sphingomyelin^51^. In the case of Venetin-1, a significant decrease in binding to sphingomyelin was observed. It may suggest loss of the ability to form the full pore characteristic of this protein or the formation of non-functional oligomers during the heating of the raw coelomic fluid. Venetin-1 can still interact with SM, but the interaction is much weaker. This is also visible in the case of POPC, which imitates mammalian membranes. DVr exerts a weaker effect than SM, but the interaction in the case of Venetin-1 is even weaker. Identification of proteins from the recovery fraction revealed lysenin-related protein 2 as a lipid-binding protein for both tested preparations. LRP-2 exhibits 89% identity and 94% similarity to the lysenin sequence, and literature data indicate that LRP binds to SM and induces hemolysis of red blood cells, likewise lysenin^51^. Structurally, they share putative N-glycosylation and N-myristoylation sites. The results show that the interaction of Venetin-1 with lipids is based on LPR2 but not on lysenin.

Venetin-1 lost its pore-forming activity or deactivation of the pore-forming activity took place. This was confirmed by the lack of hemolytic capacity of Venetin-1 towards red blood cells and demonstrated significant reduction of Venetin-1 toxicity against healthy cells and the absence of hemolysis of RBC, compared to the DVr preparation. The DVr fraction degraded RBC, while their incubation with Venetin-1 did not alter these cells and did not cause release of their contents (data not shown in the manuscript). Thus, the interaction of monomeric or oligomeric forms with the membrane surface without formation of pores and membrane perforation may be analogous in cancer cells. The pore formation process consists of three main steps in contact with the membrane: binding of soluble monomers to the membrane, assembly/oligomerization, and full incorporation of the pore form into the lipid membrane^52,53^. Venetin-1 binds monomers or possibly soluble oligomers to the membrane, but the other two steps appear to be somehow inhibited.

According to the current state of medical knowledge, there is still a need to develop new pharmaceuticals that, alone or synergistically with other drugs, will act effectively against non-small cell lung cancer. Lung cancer is one of the neoplasms with a very poor prognosis since it is often diagnosed too late. The basis for the treatment of most cancers is combination therapy, i.e., the use of surgery techniques, radiotherapy, and chemotherapy. The type of therapy depends on the tumor stage and patient's overall efficiency. During a cancer disease and chemotherapy, a fungal infection may develop in the patient as a serious complication. In this case, it is very important to know both the activities of anti-cancer cytostatics and their interactions with antifungal medicines. Chemotherapeutic agents used in oncology with antifungal properties belong to different groups of cytostatic drugs. These include platinum derivative antimetabolites, DNA topoisomerase inhibitors, and estrogen receptor modulators. Venetin-1, previously described as a protein-polysaccharide fraction, showed effective antifungal activity against *C. albicans* with no endotoxicity or cytotoxicity to normal human skin cells^54^.

In addition to the anti-tumor activity against A549 non-small cell lung cancer cells^29,55^, the fraction has a cytotoxic effect on colon cancer cells^56,57^. In addition, it has been shown that Venetin-1 activates human macrophages in vitro and causes secretion of pro-inflammatory cytokines (messenger proteins): interleukin 6 (IL-6) and interleukin 1β (IL-1β) by these cells. The invention was patented by the Polish Patent Office^58^. Venetin-1 can be used as a component of a preparation increasing resistance to infections (e.g., an immunostimulatory drug) or locally enhancing the immune response (e.g., as an adjuvant added to bacterial or viral vaccines). Since studies have shown that Venetin-1 exhibits direct antitumor activity, the immunostimulatory effect shown suggests the possibility of using Venetin-1 in cancer patients to restore the proper functioning of the immune system and fight cancer or to supplement immunodeficiency after chemotherapy.

The search for an active factor with antimicrobial and antitumor activity in the earthworm *D.* *veneta* has been conducted by our research team for over 10 years. In the first stage, these studies resulted in isolation of the symbiotic bacterium *Raoultella ornithinolytica* from the gastrointestinal tract of this species. The extracellular metabolites of this bacterium produce antibacterial, antifungal, and anticancer compounds^59–63^. Separation carried out by means of chromatographic methods is necessary to isolate the active agent. It was difficult to obtain a sufficient amount of the test substance, and the high cost of separation was another limitation.

The next step was to try to isolate the active compound from the coelomic fluid. This stage of research was successful and yielded a large amount of the active substance with cheap methods. Separation by dialysis was applied after incubation at elevated temperature to eliminate cytotoxicity. In this case, the only limitation is the number of individuals that can be freely adjusted.

The method of Venetin-1 acquisition is cheap, quick to carry out at any time, and suitable for commercial use. Currently, we have obtained the permission of the bioethics committee to conduct research on a mouse model, and the dose that will effectively stimulate the animal's immune system will be determined in the near future. The preparation will be administered intraperitoneally by injection, as it seems to be the best way to avoid loss of the preparation and to maintain the effective dose. After determining the immumodulating properties, Venetin-1 will be injected to animals with lung cancer to determine the inhibitory effect.

The anti-cancer effect of active nanoparticles from *D. veneta* on A549 lung cancer cells, confirmed by the present research, may allow the use of a safe and effective drug for lung cancer. The development of such a drug would be a breakthrough in the therapy of this disease.

## Materials and methods

### Earthworms

*Dendrobaena veneta* earthworms were grown in laboratory conditions at the Department of Immunobiology of the Maria Curie-Skłodowska University (Lublin, Poland). The invertebrates were kept in 3 L containers filled with compost soil at a temperature of 20 °C in complete darkness. The earthworms were fed with boiled vegetables and green tea leaves. The diet was enriched with lignin cellulose, which is necessary to produce cocoons.

### Extraction of Venetin-1 from coelomic fluid

Adult earthworms were kept on moist lignin for 24 h to cleanse the intestine. Then, the coelomic fluid (CF) was collected from groups of 10 earthworm individuals by mild electrostimulation (4.5 V). CF was harvested in 0.9% NaCl and centrifuged at 2500×*g* for 10 min at 4 °C to remove coelomocytes. This yielded a cell-free supernatant, which was sterilized by filtration through Millipore filters with a pore size of 0.22 µm. The cell-free CF was incubated for 10 min at 70 °C to eliminate cytotoxic properties. Next, it was transferred to a cellulose membrane bag with a cut-off of 12–14 kDa. The samples were dialyzed in water for 24 h at 4 °C. After the dialysis, Venetin-1 was lyophilized. The preparation was stored at -20 °C. The protein concentration was estimated in the lyophilized samples using the Bradford method (Bio-Rad, USA)^64^. Non-heated and non-dialyzed coelomic fluid (raw coelomic fluid—DVr) was used for proteomic analyses.

### Cell culture

Human bronchial epithelial cells (BEAS*-*2B) and lung adenocarcinoma epithelial cells (A549 cells) were cultured in 100-mm polystyrene tissue culture dishes in RPMI 1640 medium (Gibco, Invitrogen, USA) containing 8% FBS (Gibco, Invitrogen, USA) and 5% penicillin/streptomycin (Gibco, Invitrogen, USA) in a humidified incubator at 37 °C, 5% CO_2_, and 95% air. The cells were routinely passaged by trypsinization and subcultured at an initial plating density of 2 × 10^5^ for 72 h. The BEAS-2B cells were provided for the research by Dr Dorota Ścieglińska from the Maria Skłodowska-Curie Memorial Cancer Center and Institute of Oncology (Gliwice, Poland). The A549 cells came from the cell culture of the Medical University in Lublin (Poland).

### MTT assay

The methyl thiazol tetrazolium (MTT) assay was used to determine the cytotoxicity of Venetin-1 towards the BEAS-2B and A549 lung cancer cells. At first, the cells were seeded in a 96-well plate at a density of 1 × 10^4^ cells/well and allowed to adhere at 37 °C in a CO_2_ incubator for 24 h. The following day, the medium was replaced with a fresh one supplemented with various concentrations of Venetin-1: 15.6, 31.3, 62.5, 125, 250, and 500 µg/mL. Each 96-well plate was set up with control (cells-only) and zero-adjustment (medium only) groups. The assay was performed in triplicates. After 24, 48, and 72 h, the culture medium was replaced with a fresh one, and the cells were incubated with 20 µL of MTT (Invitrogen, CA USA) working solution (5 mg/µL) at 37 °C for 4 h. The supernatant was then removed, and purple formazan crystals were solubilized in 100 μl/well of dimethyl sulfoxide (DMSO) followed by incubation (1 h). The absorbance of the formazan solution was measured at 550 nm with a plate reader Victor 1420 multilabel counter (PerkinElmer, Inc. Waltham, MA USA). The cell viability percentage was calculated using the following formula: [Cell viability (%) = (experimental group OD − zero adjustment group OD)/(control group OD − zero adjustment group OD) × 100%. Cell inhibitory ratio (%) = 1 − cell viability (%)]. Each experiment was repeated three times.

### Annexin V and PI staining for apoptosis detection

For assessment of cell apoptosis, the A549 cells were seeded onto 6-well microtiter plates at a density of 2 × 10^5^ cells/mL and grown for 24 h. Next, the culture medium was replaced with a fresh one (control cells) or the cells were exposed to 125 µg/mL of Venetin-1 for 72 h. To determine the percentage of cells within the population that were actively undergoing apoptosis, an eBioscience™ Annexin V-FITC Apoptosis Detection Kit (Invitrogen, USA) was used according to the manufacturer’s instructions. In brief, the harvested cells (~ 5 × 10^5^ /mL) were resuspended in 200 µL binding buffer (1x), and 195 µL of the cell suspension was mixed with 5 µL Annexin V- FITC following 10-min incubation at room temperature. Next, the cells were washed in 200 µL of binding buffer (1x), resuspended in 190 µL of binding buffer (1x), and stained with 10 µL of propidium iodide (PI) (20 µg/mL). After 15-min incubation in the dark at room temperature, the cells were analyzed on a Guava easyCyte flow cytometer (Merck, Germany). The cells were analyzed using Merck Millipore's InCyte™ software. 10 000 events were analyzed for each sample, and the results were expressed as a percentage of cells in each category. Three independent experiments were performed.

### Cell cycle analysis

Cell cycle progression was determined using PI. The A549 cells were prepared as described above for the assessment of cell apoptosis. After 72 h, the cells were harvested, washed with PBS, and after centrifugation (300*g*, 6 min, 4 °C) fixed with ice-cold 70% ethanol at 4 °C overnight. Next, the cells were washed three times with PBS, adjusted to ~ 1 × 10^6^ cells, and stained with FxCycle™ PI/RNase Staining Solution (Thermo Fisher, USA) according to the manufacturer’s instructions (15–30 min at 37 °C). The samples were analyzed on a Guava easyCyte flow cytometer (Merck, Germany). 10,000 events within this gate were acquired per sample.

### Determination of the concentration of caspases

The study involved cultures of normal BEAS-2B cells and A549 lung cancer cells. The cultures were carried out in standard conditions (37 °C, 5% CO_2_ and 90% humidity) both without and with the addition of Venetin-1. Human CASP ELISA Kits were used to determine the concentration of caspases (FineTest, Wuhan Fine Biotech Co., Ltd., China). The caspase concentration was determined using ELISA in accordance with the manufacturer's manual. Absorbance values were read at 450 nm using a microplate reader (BioRad, USA).

### Optical microscopy analysis of Venetin-1 action on A549 cells

The A549 cancer cells, both control and incubated with Venetin-1 for 72 h (at the concentrations of 62.5 and 125 µg/mL), were imaged using a Zeiss Axiovert 40CFL microscope (Carl Zeiss, Germany). Changes in their structure were also documented with the DIC (differential interference contrast) function. The morphology of the cells was visualized at 60× magnification using an Olympus BX61 microscope (Olympus Corporation, Japan).

### Visualization of apoptotic cells by fluorescence microscopy

Changes in the A549 tumor cell morphology characteristic of apoptosis were assessed with the use of fluorescence microscopy using a mixture of Hoechst 33342 fluorochrome and PI (Sigma, Germany). The control and Venetin-1-treated cells were imaged with a Zeiss LSM 5 Pascal (Carl Zeiss, Germany) microscope at emission wavelengths of ~ 460 nm and > 575 nm, respectively. The staining mixture at a concentration of 2.5 μl/mL was added to the cell culture and the preparations were incubated for 5 min at 37 °C in the dark. Apoptosis was evidenced by the bright blue fluorescence of the nuclei of intact or fragmented cells; cells with pink nuclei were identified as necrotic.

### Scanning electron microscopy (SEM)

After the incubation with Venetin-1, the non-small lung carcinoma A549 cells were observed by SEM. First, they were fixed with 4% glutaraldehyde in 0.1 M phosphate buffer at pH 7.0. Subsequently, the cells were mounted on slides and stained with a 1% OsO_4_ solution for 1 h, followed by dehydration in a series of acetone solutions (15%, 30%, 50%, 70%, 100%). The preparations were dried in a desiccator using silica gel for 24 h and gold-plated using a K550X coater (Quorum Technologies, United Kingdom). The fixed cells were analyzed using a Tescan Vega 3 scanning electron microscope (Tescan, Czech Republic).

The structure of Venetin-1 was observed and documented with a Quanta 3D FEG scanning electron microscope (FEI, Japan). A lyophilized preparation, which had not been subjected to standard dehydration and gold sputtering, was used for microscopic observations. This procedure allowed visualization of the active substance in its natural shape.

### Atomic Force Microscopy (AFM)

The surface of the control cancer cells and the Venetin-1-treated cells was analyzed by AFM. Measurements were performed using a NanoScope V AFM microscope operated in the PeakForce Quantitative Nanomechanical Mapping mode with MultiMode 8 (Bruker, Veeco Instruments Inc.). It was equipped with NanoScope 8.15 software. The local modulus of elasticity was determined using the Derjaguin-Muller-Toporov (DMT) modulus. The nominal spring rate of the RTESPA probe (Bruker, USA) (silicone tip on a nitride lever) was 40 N/m. Young’s modulus was determined for a selected area of the A549 cell surface. The samples were prepared as described for SEM. Young’s modulus was determined for 20 fields in each sample (dark areas of low rigidity and bright areas of high rigidity). The data were analyzed statistically.

### Proteomic analysis

#### Protein separation and digestion

Separation of proteins from Venetin-1 and raw coelomic fluid (DVr) was carried out with the development of the automatic SageElf system (Sage Science, Beverly, MA, USA) in a 3% SDS-Agarose gel cassette (ELP3010). It allows separation of proteins in the 10–300 kDa range. All the necessary solutions (Loading buffer) and the marker were supplied with the cassettes in the set. The sample was dissolved in a loading solution to obtain a concentration of 200 mg protein in 26 ml. Then, 4 ml of 0.5 M DTT was added to the sample and the whole mixture was heated for 6 min at 85 °C. After cooling, 10 ml of the loading solution with fluorescent Marker-03 was added to the sample, and the sample was mixed. The proteins prepared in this way were introduced into a 3% SDS-Agarose cassette. The separation (size-based mode) consisted in automatic elution of proteins from the gel for 1 h 20 min and then 30 min. The system was controlled by SageElf software version 1.08. It yielded 12 fractions of the preparation, which were then digested using a standard FASP procedure and trypsin (Trypsin Gold, Promega)^27,65^. Fractions obtained from two runs were combined for one FASP procedure. Final cleanup was carried out according to the Stage Tips procedure^66^.

#### Mass spectrometry analysis

Spectrum registration was performed on a TripleTOF 5600+ (Sciex Framingham, MA, USA) mass spectrometer connected to a chromatography system, the Ekspert MicroLC 200 Plus System (Eksigent, Redwood City, CA, USA). All chromatographic separations were performed on the ChromXP C18CL column (3 µm, 120 Å, 150 × 0.3 mm). For each sample, the chromatographic gradient for each MS run was 11–42.5% B (solvent A: 0% aqueous solution, 0.1% formic acid; solvent B: 100% acetonitrile, 0.1% formic acid) for 60 min. The whole system was controlled by SCIEX Analyst TF 1.7.1 software. Measurements for the spectral library were acquired in the data-dependent acquisition (DDA) mode. Each cycle of the applied DDA method comprised accumulation of precursor spectra in 100 ms in the range of 400–1200 m/z followed by accumulation of top 20 precursor's product ion spectra in 50 ms in the range of 100–1800 m/z, resulting in a total cycle time of 1.15 s. Formerly fragmented precursor ions were dynamically excluded.

PeaksStudio XPRO software (Bioinfor, Canada) was used for database search. Protein identification was carried out based on the reviewed Annelida database (05.04.2022, Uniprot) with the following parameters: filter charge 2–8, error tolerance for precursor mass—25 ppm, fragment ion tolerance 0.5, maximum missed cleavages per peptide—three fixed modifications—carbamidomethylation, variable—Met oxidation, 1% FDR for peptides and proteins. The final analyses were performed based on the Spider file result. The obtained data were deposited in the Pride repository^67^ under the identifier.

#### Preparation of lipids

For the SPR experiments, 3 mM molar sphingomyelin (SM) and 1-palmitoyl-2-deoylphpsphatydilcholine (POPC) liposome stock solutions were prepared. The liposome solution was prepared as described previously^68^. Briefly, POPC (Avanti Polar Lipids, USA) and sphingomyelin (chicken egg; Avanti Polar Lipids, USA) lyophilized powder were suspended in PBS buffer (Sigma Aldrich, USA). The phospholipid suspension was subjected to 10 incubation cycles involving 30-min incubation in an ultrasound bath with heating (333 K) and 30-min incubation at 277 K. Next, extrusion was performed using an Avanti Mini Extruder (Avanti Polar Lipids, USA) with a 0.1 µm pore membrane. This procedure, i.e., passing the lipid solution through the extruder, was repeated eleven times. After completion of the extrusion procedure, the sample was ready for use.

#### SPR experiment

The binding of the sphingomyelin (SM) and 1-palmitoyl-2-deoylphpsphatydilcholine (POPC) lipids was performed on the L1 sensor (Cytiva, USA) using the Biacore T200 instrument (Cytiva, USA). The interaction of the proteins was analyzed after the immobilization of the lipids (SM or POPC) on the surface of the L1 sensor chip as described in the manufacturer's manual. SM was immobilized to the response level of 3659.96 RU (± 415.45 RU), and POPC was immobilized to the response level of 4995.67 RU (± 570.47 RU). Then, after blocking the sensor with a 0.5 mg/mL albumin solution, the standard manufacturer's protocol "Injection and Recovery" was employed. The protein extracts were run over the surface of the sensor chip with immobilized lipids allowing protein binding. Next, after 30-s incubation with 0.5% TFA, bound proteins were recovered in 50 µl of 50 mM NH_4_HCO_3_. Then, the samples retrieved from at least two flow cells were analyzed with MS. After all the analyses, the background reference response spectrum was subtracted from the experimental response spectrum, and the results were presented in sensorgrams.

#### Protein digestion and identification from SPR recovery fractions

Fractions collected from the SPR experiment containing proteins bound to the tested lipids were subjected to classical digestion in a solution. Before the trypsin addition, the samples were treated with a 50 mM DTT solution (45 min, 56 °C), followed by IAA (45 min in the dark). The trypsin digestion was carried out overnight at 37 °C. When the digestion was stopped by lowering the pH of the solution (1% formic acid in water), the samples were purified by StageTips and concentrated at 30 ml. The LC–MS/MS analysis and identification of proteins were carried out analogically as in the case of the Venetin-1 samples.

### Cryo-TEM analysis of Venetin-1

The Venetin-1 solution with a protein concentration of 1 mg/mL was placed in an ultrasonic chamber (Pol-Sonic, Poland) for 10 min at 40 °C. The aqueous suspension was then vitrified on a TEM mesh with a punched carbon film (Quantifoil R 2/2; Quantifoil Micro Tools GmbH, Großlöbichau, Germany). The mesh was previously activated for 15 s in oxygen plasma using a Femto device (Diener Electronic, Ebhausen, Germany). A 3-µL sample was applied to the mesh. Thereafter, blotting with filter paper and instant freezing in liquid ethane using a Vitrobot Mark IV blower (FEI Company, Hillsboro, Oregon, USA) were performed. Glazed samples were kept in liquid nitrogen until placing in a Cryo-TEM Gatan 626 holder (Gatan Inc., Pleasanton, USA). Cryogenic Transmission Electron Microscopy (cryo-TEM) images were obtained using a Tecnai F20 X TWIN microscope (FEI Company, Hillsboro, Oregon, USA) equipped with a Field Emission Cannon (FEG) operating at an acceleration voltage of 200 kV. An Eagle 4 k HS camera (FEI Company, USA) was used for image recording and TIA software (FEI Company, USA) was used for processing.

### X-ray photoelectron spectroscopy (XPS) analysis of Venetin-1

The chemical studies of Venetin-1 were performed using the XPS electron spectroscopy method. The analyses were performed in the Prevac UHV multi-chamber analytical system (Prevac, Poland). After mounting on the molybdenum support, the tested Venetin 1 samples were degassed at room temperature to a constant high vacuum of ~ 5 × 10^–8^ mbar in the UHV system loading lock. The XPS analysis was performed after the sample was introduced into the analytical chamber of the UHV system. A monochromatic AlKα radiation source served as a photoelectron excitation source. The photoelectrons were excited by X-ray radiation with a characteristic line AlKα and energy of 1486.7 eV generated by a VG Scienta SAX 100 lamp with an aluminum anode together with a VG Scienta XM 780 monochromator.

Survey spectra were performed in a wide range of binding energies, using the following analyzer parameters: operating mode—sweeping, 200 eV transition energy, measured range of photoelectron binding energy 0–1200 eV, measurement step of 0.5 eV, collection time in a single step—0.2 s, number of iterations—4.

After the survey spectrum was performed, the spectra in a narrow range of binding energies were also made for carbon, oxygen, nitrogen, phosphorus, and sulfur. The tests were performed with the following analyzer parameters: operating mode—sweeping, 50 eV transition energy, measurement step—0.1 eV, collecting time in a single step—0.667 s.

For quantitative calculations, the obtained spectra were processed with Casa XPS software. The X-axes corresponding to the bond energy were calibrated using the C1s aliphatic carbon peak. The binding energy corresponding to this peak was assumed to be EB = 285.0 eV.

### Dynamic light scattering (DLS) domain-specific characterization of Venetin-1

To characterize the size of the Venetin-1 microparticles, we performed a DLS experiment using the Prometheus Panta (NanoTemper Technologies GmbH, Germany) instrument. The samples were loaded into eight capillaries acting as replicas where the parallel analysis revealed the homogeneity of the microparticles.

## Supplementary Information

Supplementary Information 1.

Supplementary Information 2.

Supplementary Information 3.

Supplementary Information 4.

Supplementary Information 5.

## Data Availability

Data obtained from proteomic analyzes have been deposited in the PRIDE repository and are available under the accession number PXD034132.
