# Supplementary material for: Novel Venetin-1 nanoparticle from earthworm coelomic fluid as a promising agent for the treatment of non-small cell lung cancer
Source: Sci Rep. 2022 Nov 2;12:18497. doi: 10.1038/s41598-022-21665-8 (PMC9630273; doi:10.1038/s41598-022-21665-8)
Supplement: Supplementary file 1 — Supplementary Information 1. [file 41598_2022_21665_MOESM1_ESM.docx]

**Supplementary Figure S1**

Activity of Venetin-1 in BEAS-2B cells assessed with Annexin V/PI staining assay. Panel A shows representative dot plots of A549 control cells (without incubation with Venetin-1) after double staining with Annexin V-FITC and PI. Panel B shows the effects of incubation with Venetin-1 (125 µg/mL) for 72 h on cell apoptosis in BEAS-2B cells. The cells were classified as viable cells (lower left square), early apoptotic cells (lower right square), late apoptotic cells (upper right square), and necrotic cells (upper left square). These are representative images of three independent experiments. Venetin-1 nanoparticle does not affect the number of living cells and rate of necrosis, early and late apoptosis in normal lung epithelial cells in comparison with untreated cells.

**Supplementary Table S5**

Summary of the results of protein identification for the recovery fraction from the SPR experiments. Liposomes (SM, POPC) were bound on the L1 sensor, and the coelomic fluid fraction labeled as Venetin-1 and the pure coelomic fluid fraction labeled as DVr were bound to the lipids. After the complex was dissociated, the recovery fraction for both experiments was subjected to trypsin in solution digestion, MS/MS spectra were recorded, and identification was performed based on the Annelida Uniprot database in the PeaksStudio program. Table 1 shows the proteins identified, Table 2 the identified peptide sequences for these proteins for Venetin 1. Shown below is the protein sequence with the peptides identified underlined in gray. Table 3 shows the proteins identified for DVr fraction, Table 2 the identified peptide sequences for these proteins for DVr. Shown below is the protein sequence with the identified peptides underlined in gray.
