# Supplementary figures and images for: Novel Venetin-1 nanoparticle from earthworm coelomic fluid as a promising agent for the treatment of non-small cell lung cancer
Source: Sci Rep. 2022 Nov 2;12:18497. doi: 10.1038/s41598-022-21665-8 (PMC9630273; doi:10.1038/s41598-022-21665-8)

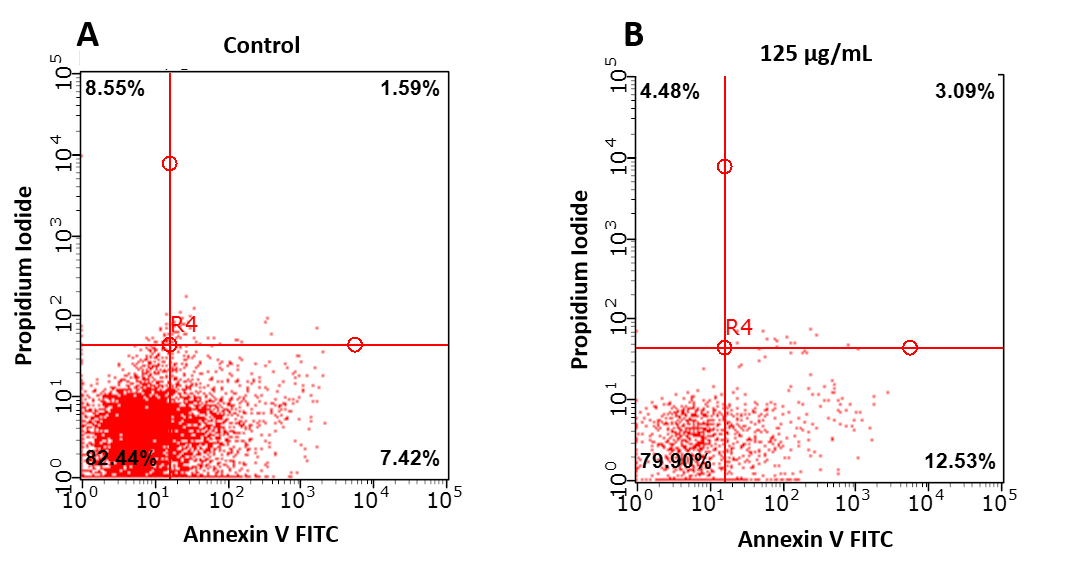

Supplement: Supplementary file 2 — Supplementary Information 2. [file 41598_2022_21665_MOESM2_ESM.tif]
