# Supplementary material for: Novel Venetin-1 nanoparticle from earthworm coelomic fluid as a promising agent for the treatment of non-small cell lung cancer
Source: Sci Rep. 2022 Nov 2;12:18497. doi: 10.1038/s41598-022-21665-8 (PMC9630273; doi:10.1038/s41598-022-21665-8)
Supplement: Supplementary file 3 — Supplementary Information 3. [file 41598_2022_21665_MOESM3_ESM.docx]

**Supplementary Table S2.** Concentrations of caspases 3, 6, 8, 9, 12, and 18 in BEAS-2B and A549 cell cultures. * p<0.05.

| Cell culture | Concentration of caspase [ng/mL] | | | | | |
| --- | --- | --- | --- | --- | --- | --- |
|  | **caspase 3** | **caspase 6** | **caspase 8** | **caspase 9** | **caspase 12** | **caspase 18** |
| BEAS-2B cells | 0,160±0.02 | 2.648±0.8 | 0,995±0.1 | 1,055±0.4 | 1.32±0.2 | 0,105±0.02 |
| BEAS-2B cells + Venetin-1 (125 µg/mL) | 0,165±0.01 | 2,828±0.7 | 1.002±0.2 | 1.218±0.2 | 1.28±0.3 | 0,098±0.01 |
| A549 lung cancer cells | 0.290±0.03 | 4,064±0.8 | 0,785±0.1 | 1,506±0.3 | 6.35±0.8 | 0,102±0.02 |
| A549 lung cancer cells + Venetin-1 (125 µg/mL) | 0,726±0.0.05* | 5,828±0.9* | 0.998±0.1* | 1.988±0.4* | 8.80±0.8* | 0,199±0.02* |
